# Transcriptomic profiling of *Burkholderia phymatum* STM815, *Cupriavidus taiwanensis* LMG19424 and *Rhizobium mesoamericanum* STM3625 in response to *Mimosa pudica* root exudates illuminates the molecular basis of their nodulation competitiveness and symbiotic evolutionary history

**DOI:** 10.1186/s12864-018-4487-2

**Published:** 2018-01-30

**Authors:** Agnieszka Klonowska, Rémy Melkonian, Lucie Miché, Pierre Tisseyre, Lionel Moulin

**Affiliations:** 1 IRD, Cirad, University of Montpellier, IPME, Montpellier, France; 2 IRD, UMR LSTM, Campus de Baillarguet, Montpellier, France; 3 Present address: Aix Marseille University, University of Avignon, CNRS, IRD, IMBE, Marseille, France

**Keywords:** Symbiosis, Transcriptome, Beta-rhizobia, Alpha-rhizobia, RNA-seq

## Abstract

**Background:**

Rhizobial symbionts belong to the classes Alphaproteobacteria and Betaproteobacteria (called “alpha” and “beta”-rhizobia). Most knowledge on the genetic basis of symbiosis is based on model strains belonging to alpha-rhizobia. *Mimosa pudica* is a legume that offers an excellent opportunity to study the adaptation toward symbiotic nitrogen fixation in beta-rhizobia compared to alpha-rhizobia. In a previous study (Melkonian et al., Environ Microbiol 16:2099–111, 2014) we described the symbiotic competitiveness of *M. pudica* symbionts belonging to *Burkholderia*, *Cupriavidus* and *Rhizobium* species.

**Results:**

In this article we present a comparative analysis of the transcriptomes (by RNAseq) of *B. phymatum* STM815 (BP), *C. taiwanensis* LMG19424 (CT) and *R. mesoamericanum* STM3625 (RM) in conditions mimicking the early steps of symbiosis (i.e. perception of root exudates). BP exhibited the strongest transcriptome shift both quantitatively and qualitatively, which mirrors its high competitiveness in the early steps of symbiosis and its ancient evolutionary history as a symbiont, while CT had a minimal response which correlates with its status as a younger symbiont (probably via acquisition of symbiotic genes from a *Burkholderia* ancestor) and RM had a typical response of Alphaproteobacterial rhizospheric bacteria. Interestingly, the upregulation of nodulation genes was the only common response among the three strains; the exception was an up-regulated gene encoding a putative fatty acid hydroxylase, which appears to be a novel symbiotic gene specific to *Mimosa* symbionts.

**Conclusion:**

The transcriptional response to root exudates was correlated to each strain nodulation competitiveness, with *Burkholderia phymatum* appearing as the best specialised symbiont of *Mimosa pudica*.

**Electronic supplementary material:**

The online version of this article (10.1186/s12864-018-4487-2) contains supplementary material, which is available to authorized users.

## Background

Rhizobia are a functional group of bacteria able to develop a nitrogen-fixing symbiosis with legumes. Symbiotic associations between legumes and rhizobia usually begin with an exchange of diffusible signals among which (iso)flavonoids present in the root exudates are responsible for the production and secretion of bacterial lipochitooligosaccharides (LCOs), known as Nod factors (NF). The recognition of NF by the roots leads to the formation of the root nodule, wherein bacteria are able to fix atmospheric nitrogen to sustain plant growth in exchange for carbon –containing metabolites [1]. The biosynthesis of NF and nitrogen fixation enzymes are encoded by *nod* and *nif* genes respectively [2].

Rhizobial species are mainly distributed among four genera of Alphaproteobacteria (*Rhizobium*, *Sinorhizobium*, *Mesorhizobium*, *Bradyrhizobium;* and a few species in seven other genera) and two genera of Betaproteobacteria (*Burkholderia*, *Cupriavidus*) leading to the proposal of the terms alpha- and beta-rhizobia to distinguish both classes of symbionts [3]. The discovery of beta-rhizobia in 2001 changed dramatically our perspectives on the genetic backgrounds required to host the ability to form symbioses with legumes by extending this possibility to a new subclass of Proteobacteria. If beta-rhizobia appeared controversial at the time of their discovery, it is now well established that *Burkholderia* species are the main symbionts of several endemic species of the *Mimosoideae* and *Papilinoideae* legume subfamilies in Brazil and South Africa [4–7], which raises questions about the origin of symbiosis and performance of these new symbionts compared to alpha-rhizobia. Many symbiotic species of *Burkholderia* (recently rearranged in the *Paraburkholderia* genus [8]) have been described from mimosoids: *B. phymatum* [9], *B. tuberum* [10], *B. mimosarum* [11], *B. nodosa* [12, 13], *B. sabiae* [14], *B. symbiotica* [15], *B. diazotrophica* [16], *B. piptadeniae* and *B. ribeironis* [17], while *Cupriavidus* symbionts (*C. taiwanensis, C. necator, C. pinatubonensis*) were detected mostly in invasive *Mimosa* species (*M. pudica*, *M. pigra*, *M. diplotricha*) in South, Central and North America [10, 18, 19], in Asia (China, India, Taiwan, Philippines, New Caledonia, Papua New Guinea) [20–24], but also in native *Mimosa* and *Parapiptadenia* species in Uruguay [25, 26].

Diversity studies of alpha-rhizobia showed incongruence between rhizobial house-keeping genes and symbiotic *nod* genes phylogenies. Phylogenetic analyses have shown that the different rhizobial lineages diverged well before the appearance of legumes [27], so that their bacterial nodulation capacity would have been acquired substantially after the diversification of rhizobia in one or several lineages, and was then spread by lateral gene transfer among the different genera and species [28, 29]. For the Mimosoid *Burkholderia* legume symbionts, the scenario seems to be different as their *nod* gene phylogenies mirror that of the *Burkholderia* species (based on their 16S rRNA and housekeeping genes), which has led several authors to hypothesise an ancient transfer of nodulation genes to a burkholderia ancestor followed by vertical transmission and coevolution with endemic host species [30]. In the case of *Cupriavidus*, the nodulating species were proposed to be recent symbionts compared to *Burkholderia* based on phylogenetic and genomic analyses [31], but in the light of recent findings and analyses (*nodC* gene phylogeny, symbiotic specificity) its origin and evolution may be more complex [5].

*Mimosa pudica* is a legume that has the rare ability to be nodulated naturally by both alpha (*Rhizobium* spp.) and beta-rhizobia (*Burkholderia*, *Cupriavidus*). Diversity studies showed that proportions between beta- and alpha-rhizobia hosted in nodules of *M. pudica* vary significantly in different geographical locations. For example, *Burkholderia* were found to be predominant in nodules of *M. pudica* in Barro Colorado Island and in French Guiana [10, 32], whereas the proportions of *Burkholderia* and *Cupriavidus* symbionts were more equal in Costa Rica, China and India [18, 20, 24], and finally *Cupriavidus* was found to be predominant in nodules of *M. pudica* in Taiwan and in New Caledonia [23, 33]. Several parameters can help explain the proportions of symbionts in nodules of *M. pudica* in worldwide diversity studies, but soil characteristics, especially the presence of combined nitrogen affect strain competitiveness [34]. In a previous study [35], we made a large survey of strain nodulation competitiveness between *Burkholderia*, *Cupriavidus* and *Rhizobium* species and showed that *B. phymatum* and *B. tuberum* sv. *mimosae* are the most competitive species on *Mimosa pudica*, but *Cupriavidus taiwanensis* could compete with *Burkholderia* when tested on specific *M. pudica* genotypes (on variety *unijuga* from Taiwan where *Cupriavidus* dominates in nodules of this variety). In the case of *Rhizobium* species, all species were poorly competitive, despite the fact that *R. mesoamericanum* are frequently found in diversity studies, although in small proportions, such as in Costa Rica [18], Mexico [36], New Caledonia [23] and French Guiana [10], which raises questions about their capacity to be maintained as *M. pudica* symbionts. The only case of a *Rhizobium* species dominating in the nodules of *M. pudica* is *R. altiplani* in central Brazil [37].

Several molecular factors linked to nodulation competitiveness have been described in rhizobia, including i) the nodulation gene set [38], ii) rhizobial motility [39] and specific adhesiveness to the plant roots [40], iii) adjustment of rhizobial cell-surface characteristics (EPS and LPS) [41], iv) induction of type III or type IV secretion system expression [42] and v) induction of pSym conjugal transfer genes [43]. Until now, all these studies have been performed on alpha rhizobia, which leaves many questions about the specific adaptations that might be found in beta-rhizobia which could explain their competitiveness superiority over alpha-rhizobia in *Mimosa* species. Beyond the activation of *nod* and *nif* genes, no studies have been so far conducted concerning the mechanisms of competition for nodulation or on the symbiotic process at the molecular level in beta-rhizobia.

In this study, we investigated the molecular bases of competitiveness and symbiosis between three representative and well-studied *M. pudica*-nodulating symbiotic strains of *Burkholderia (B. phymatum* STM815), *Cupriavidus* (*C. taiwanensis* LMG19424) and *Rhizobium* (*R. mesoamericanum* STM3625) by studying their transcriptomic response to root exudates as a mimicking condition of early symbiotic events. Our aim was to analyse the different molecular strategies of each strain (alpha- or beta-rhizobial), to nodulate *Mimosa pudica* in order i) to reveal commonalities and differences in the molecular dialogues of alpha and beta-rhizobia nodulating the same host, and ii) to reveal the factors underpinning the superiority of *B. phymatum* in terms of competitiveness compared to the other two symbionts.

## Results & discussion

### Symbiotic and genomic features of the three model rhizobia

Three symbionts of *M. pudica* belonging either to alpha-rhizobia (*R. mesoamericanum* STM3625 (RM)) or to beta-rhizobia (*B. phymatum* STM815 (BP) and *C. taiwanensis* LMG19424 (CT)) were used in this transcriptomic study. The symbiotic features (efficiency, nodulation competitiveness) and genome characteristics of each strain are given in Table 1. BP is characterized by a larger genome compared to the two other rhizobia (with an additional 2.2 Mb). The number of chromosomes and plasmids varies among the strains, but all harbor a symbiotic plasmid of similar size (500 to 600 kb). The core genome is composed of 1687 genes (Additional file 1: Figure S1), and, as expected, BP and CT have more additional orthologs in common with each other (1466 among the two Betaproteobacteria) than with RM. BP has 4651 specific genes compared to the other two strains. The symbiotic plasmids in all three rhizobia shared poor syntenies (Additional file 1: Figure S2), with almost only the *nod* and *nif* gene regions being syntenic.

**Table 1:** Strains with symbiotic and genomic features

Strain	Symbiotic features	G size (Mb)	Chromosomes (Mb)	Plasmids (size in kb)
BP STM815	Nodulates *Mimosa* species; highly competitive on *M. pudica.*	8.6	2 chr (3.4 and 2.6)	1 mgpl (1.9) and a pSym (0.59)
CT LMG19424	Nodulates *Mimosa pudica*; poorly competitive for nodulation on Brazilian ecotypes.	6.4	2 chr (3.4 and 2.5)	1 pSym (557)
RM STM3625	Nodulates *Mimosa pudica*. Poorly competitive compared to BP and CT on *M. pudica.*	6.4	1 chr (4.1)	1 mgpl (1.5) and 3 pl (0.55 (pSym), 0.108, 0.087)

*Abbreviations: BP Burkholderia phymatum*, *CT Cupriavidus taiwanensis*, *RM Rhizobium mesoamericanum*, *G* genome, *mgpl* megaplasmid, *pl* plasmid, *chr* chromosome, *pSym* symbiotic plasmid. Genome references: Moulin et al., [144] (BP), Amadou et al., [31] (CT), Moulin et al., [145] (RM). Reference for nodulation competitiveness: Melkonian et al., [35]. Experiments from this study were performed with similar commercial seeds from the Melkonian et al. study. Abbreviations: *G* genome, *Mb* megabase, *chr* chromosome, *mgpl* megaplasmid, *pl* plasmid, *pSym* symbiotic plasmid

### Analysis of RNAseq data and their validation by RT-qPCR

We generated RNAseq data from triplicates of cultures of BP, CT and RM at mid-log exponential phase of growth, induced or not by Root Exudates (RE) of *Mimosa pudica*. The general characteristics of RNAseq data are presented in Additional file 1: Figure S3. The number of reads that passed the initial quality filter (see Mat&Methods) varied from 32 to 111 million. Between 98.16 and 99.78% of reads mapped at least once on each corresponding genome. After in silico depletion of remaining rRNA reads, 3.5 to 18.4 million reads corresponding to mRNA were obtained per sample, representing on average 13 (for BP and CT) to 25% (for RM) of total RNAseq reads.

**Fig. 1:**
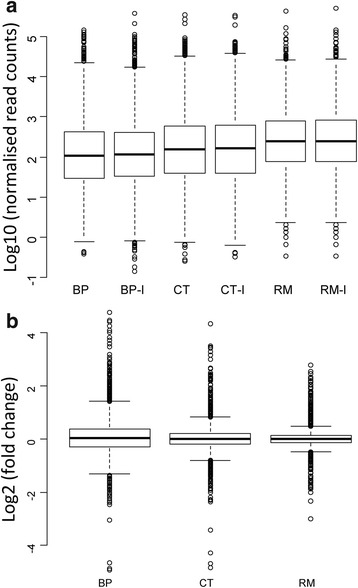
**a**-**b**. Boxplots of Read counts number (Log10) per gene per condition for each bacterium (**a**), and of Log2 (Fold Change) of gene regulation per bacterium (**b**). BP, CT and RM: RNAseq data analysis from bacteria in broth culture sampled a 4.5, 4.5 and 6 h, respectively (corresponding to mid-exponential growth phase); BP-I, CT-I and RM-I are the same as previously mentioned, but mixed with *Mimosa pudica* root exudates

Box plots of transformed log10 (Read Counts) were used to compare the distributions of our RNAseq data between conditions and between strains and to visualize the effect of normalization. As shown in Fig. 1a, the median and intra-quartile range were equal between the two conditions in each bacterium, but different between bacteria. While BP and CT data distributions were comparable, they differed from those of RM as indicated by box sizes and box plot whiskers. Also, the median reads count per CDS was higher in RM (log value 2.49; 314 reads) than in BP (log value 2.05; 112 reads) or CT (log value 2.196; 157 reads). The box plots representing the distribution of log2 fold change per strain showed further the differences in the response toward RE of the three rhizobia (Fig. 1b). The interquartile values, whisker sizes (+/− 1.5 of the interquartile values) and variance values show that the addition of RE to the culture medium had an important effect on gene expression levels of BP, but were smaller in the case of CT and had a very small effect in the case of RM.

Differentially Expressed Genes (DEG) were selected following the procedure indicated in Methods. For the selected cut-off value (pVal ≤0.01 and fold change < 0.66 (down) or > 1.5 (up)), the number of DEG was 1620, 422 and 237 for BP, CT and RM, respectively. We also compared the number of DEG depending on pVal values and fold change cut-off (Additional file 1: Figure S4), and this showed that the differences in the number of DEG genes was low when varying pVal from 0.05 to 0.001 for a same FC.

To validate the robustness of RNA-seq data, we compared them with the expression of a selection of genes analysed by RT-qPCR. Both specific (5 BP, 11 CT, 7 RM) and common DEG to all three rhizobia (13), up- or down-regulated genes, and those localized on different replicons, were chosen. The characteristics of genes tested are listed in Additional file 1: Figure S5. Two constitutively expressed reference genes (*uppS* and *hisB*) were used for qPCR normalisation. RT-qPCR values of genes plotted vs. respective RNA-seq data showed a strong correlation of gene expression between the two methods (BP: 0.82, CT: 0.77, RM: 0.92), validating our RNAseq data (Additional file 1: Figure S6).

### Transcriptomic profiles of the three model rhizobia - general overview

**Fig. 2:**
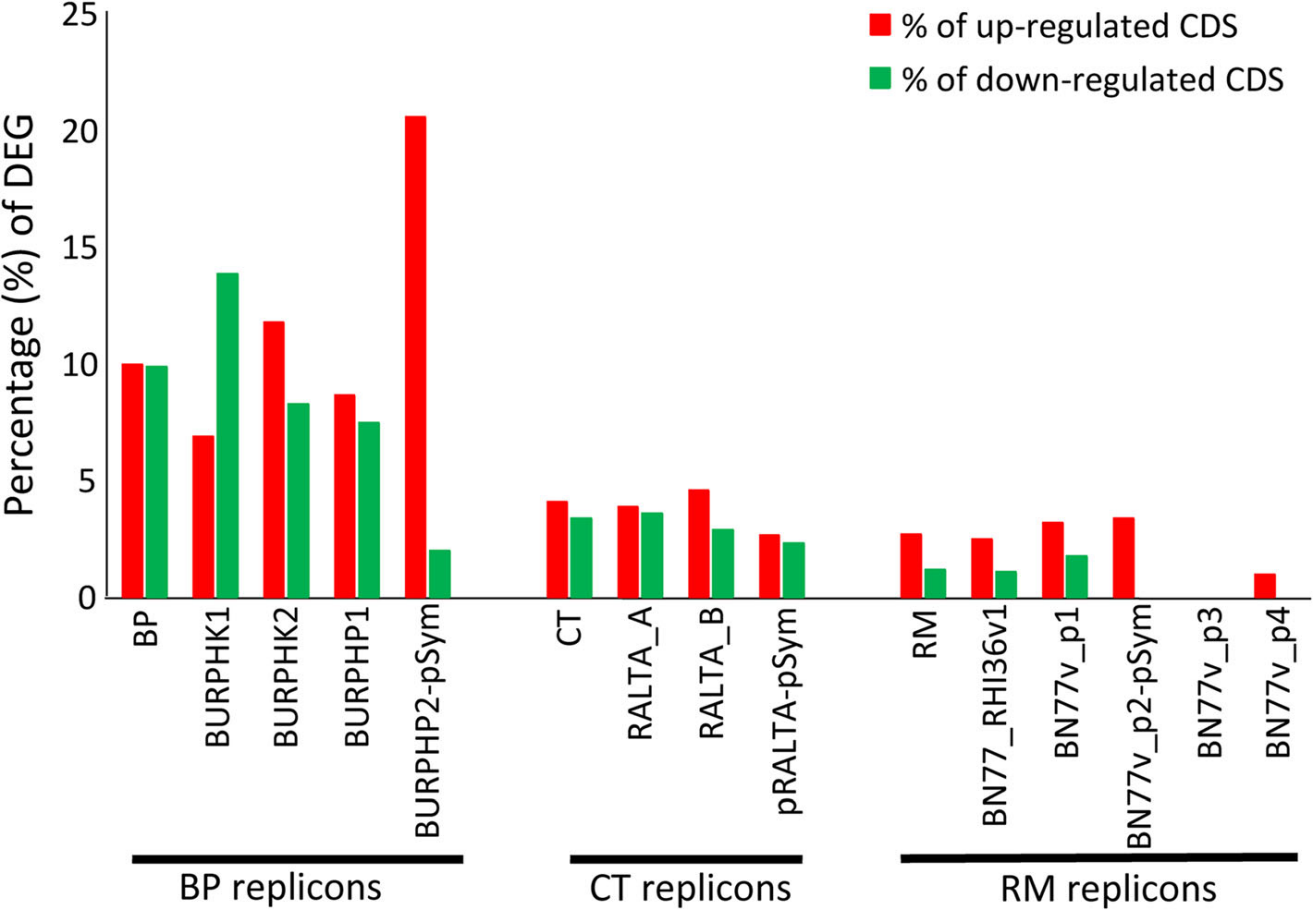
% of DEG per genomes and replicons of bacteria induced by *M. pudica* root exudates. BP (*B. phymatum* STM815), CT (*C. taiwanensis* LMG19424) and RM (*R. mesoamericanum* STM3625). Replicons names according to Table 2

The three rhizobia showed different responses to RE at mid-log growth phase. Differences were observed for the number of differentially regulated genes (Table 2), FoldChange values (Fig. 1b), and contribution of each replicon to the transcriptional response (Fig. 2). BP displayed the most complex response with 1620 differentially expressed genes which represent 19.2% of the BP genome. Moreover, BP also showed the greatest number of highly expressed genes, the highest proportion of down-regulated genes (796 down-regulated genes, representing 49.2% of all differentially expressed genes), as well as the highest Fold Change values (FC ≥ ∣4∣, not shown) (Fig. 1b). The CT and RM responses were proportionally 4- and 7-fold lower (422 and 237 differentially regulated CDS, corresponding to 7.05 and 3.62% of the CT and RM genomes, respectively). These weaker responses were not proportional to the size of bacterial genomes and should not result from differences in RNAseq data quality. The contribution of replicons to the pool of differentially expressed genes was specific for each strain (Fig. 2). The percentage of induced and repressed genes was the highest in BP representing, on average, 9% per replicon, with an exception for the pSym plasmid. The BP pSym seemed highly stimulated by the induction with RE as we detected a transcriptional induction of 20.5% (129 CDSs) and repression of 1.9% of pSym genes. In the case of CT and RM, up-regulated genes represented, on average, 3.7 and 2% per replicon, respectively. This proportion was lower for down-regulated genes representing 3 and 0.6% for CT and RM, respectively. In contrast to BP, the percentage of induced genes for the CT and RM pSyms did not differ from those of their corresponding chromosomes (Fig. 2). Indeed, only 15 and 19 genes localized on the CT and RM pSyms, respectively, were induced (Fig. 2 and Additional file 1: Figure S2).

**Table 2:** Distribution of RE-regulated genes among replicons

Genome/replicon	CDS	Up-regulated	Down-regulated	% of up	% of down
BP	8436	824	796	9.7	9.4
BURPHK1 (chr)	3301	226	429	6.8	13.0
BURPHK2 (chr)	2666	311	219	11.7	8.2
BURPHP1 (pl)	1840	158	136	8.6	7.4
BURPHP2 (pSym)	629	129	12	22.9	2.1
CT	5982	236	186	3.9	3.1
RALTA_A (chr)	3145	120	109	3.8	3.5
RALTA_B (chr)	2254	101	64	4.5	2.8
pRALTA (pSym)	583	15	13	2.6	2.2
RM	6545	167	70	2.6	1.1
BN77_v1 (chr)	4158	100	43	2.4	1.0
BN77_p1 (pl)	1540	47	26	3.1	1.67
BN77_p2 (pSym)	576	19	0	3.3	0
BN77_p3 (pl)	132	0	0	0	0
BN77_p4 (pl)	114	1	0	0.9	0
BN77_p5 (pl)	12	0	0	0	0
BN77_p6 (pl)	13	0	1	0	7.7

*BP B. phymatum* STM815 genome, *CT C. taiwanensis* LMG19424 genome, *RM R. mesoamericanum* STM3625 genome, *Chr* chromosome, *pl* plasmid, *pSym* symbiotic plasmid. Replicon names are according to their genbank annotation

**Fig. 3:**
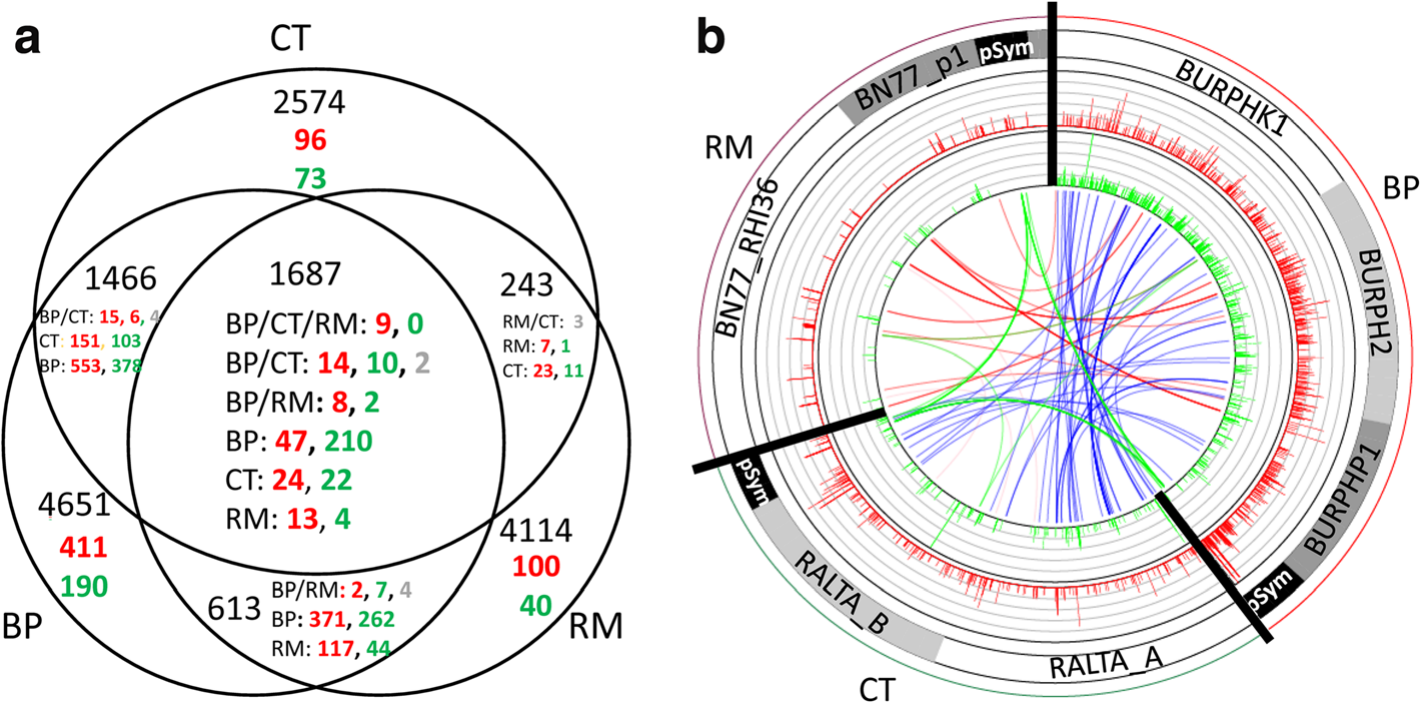
**a**-**b**. Venn diagram of DEG orthologs among the 3 rhizobia (**a**) and their circular representation (**b**). On Venn diagram (**a**), numbers in black indicate the orthologs between bacterial genomes (at intersect) or their specific gene set. Numbers in colors indicate the number of up-regulated genes (red) or downregulated genes (green) among the orthologs at each intersection of the Venn diagram. Numbers in purple indicate gene orthologs regulated in opposite direction between strains. The circular representation (**b**) was made with RCircos and represent the bacterial replicons (outer circles), their up (red) and down (green) root exudate-regulated genes (log2(fold change) values), as well as links between co-regulated orthologs (green links, between all three rhizobia; blue lines between BP and CT (beta-rhizobia); red links between RM-BP; pink lines: RM-CT). Note the high number of blue lines depicting the high number of shared regulated genes between beta-rhizobial orthologs, while few were shared by all (green links) or between RM-CT and BP-RM (pink and red links)

Among the 1687 orthologous genes constituting the core genome of the three rhizobia, 365 genes showed a differential expression in at least one of the strains (Fig. 3a). We distinguished the different categories of regulation in the center of Fig. 3a. Nine genes were similarly regulated (all up-regulated), with eight genes located on pSym (mainly *nod* genes), and one (*flgG*) on the chromosome of each bacterium. Common response of beta-rhizobia (both from core genome set and specific BP/CT orthologs) includes 51 genes (29 up-regulated; 16 down-regulated, 6 in opposite directions) distributed among all replicons in BP and in CT, while 23 genes were found to be regulated in common between BP and RM (10 up, 9 down, 4 in opposite directions) and only three genes between CT and RM (all in opposite directions) (Fig. 3a, Additional file 1: Table S1). We represented the different shared orthologs between replicons in a circular view (RCircos) in Fig. 3b. Many regulated gene orthologs were shared between BP and CT (51 blue links in Fig. 3b), while few were shared between all (9 orthologs, green links) and between BP-RM (23 red links) and RM-CT (3 pink links).

**Fig. 4:**
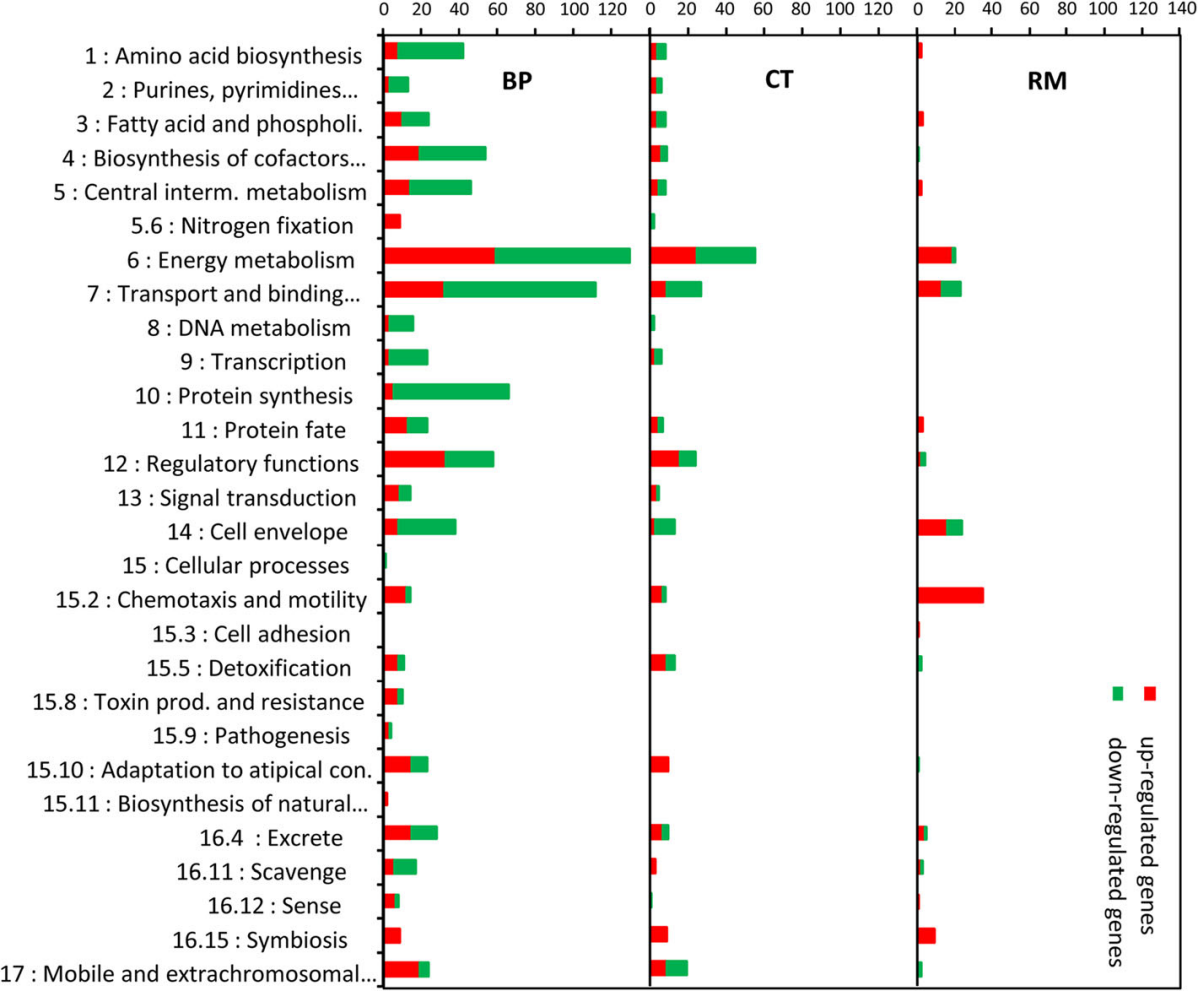
COG functional categories of differentially expressed genes after bacterial treatment by root exudates. Up-regulated COG categories are in red, downregulated in green

Concerning the functional classification of DEG, we observed differences among the number of processes affected in each rhizobial species (Fig. 4). However, similarities between rhizobia could be observed when comparing the most affected functional classes (Fig. 4) as Energy metabolism (Biop 6), Transport and binding (Biop 7) and Chemotaxis and motility (Biop 15.2) classes that reflect the typical bacterial responses to the presence of RE components/substrates [44–46]. Rhizobia showed also changes in Regulatory functions (Biop 12), Cell envelope (Biop 14), Excrete (Biop 16.4) and Symbiosis (Biop 16.5) functional classes, which can be linked to processes to interaction with the host plant. All these results suggest that BP reacted strongly to the presence of *M. pudica* RE and deeply reorganized its metabolism and physiology, while CT and RM adjusted the energy metabolism and cellular functions.

### RE-regulated functions in the three rhizobia

A general overview of the regulated functions in each rhizobium is presented in Table 3 and all expression data are compiled in Additional file 1: Table S1. In the next sections, we present an analysis of the common and specific responses of each rhizobia to the presence of root exudates.

**Table 3:**
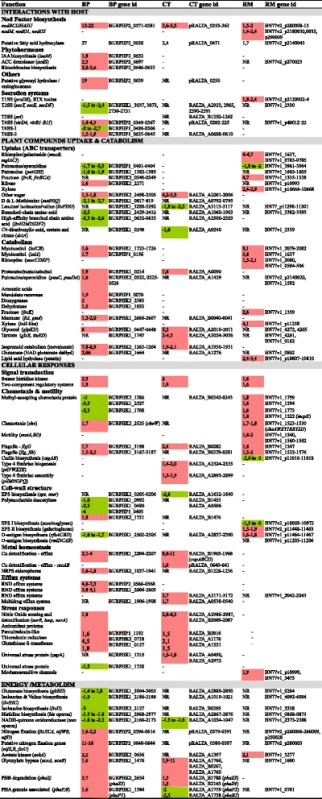
Overview of DEG functional classification in each rhizobium

For each gene functional category, a log2(fold change) is indicated and colored in red if up regulated (> 1.5) or green if down-regulated (<−1.5). *P* values of DEG are all < 0.01 (cut-off value) (see Additional file 1: Table S1 for values). The gene ID in the genome is indicated below each fold change. NR means Not Regulated by root exudates (genes involved are present but not regulated), while a minus sign (−) indicates the absence of orthologs of such genes in the genome

### Common transcriptomic responses of the three rhizobia: Nodulation genes

**Fig. 5:**
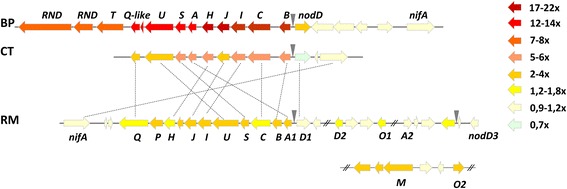
Nodulation gene operons in BP, CT and RM, colored following their regulation level (fold change) in response to root exudates at 4.5, 4.5 and 6 h post induction, respectively. Black triangles indicate the presence of a conserved nodbox (**AAT**N**GAT**TGTTTN**GAT**NNNNNN**NAT**). The regulation color scale is indicated on the right side of the figure

Nine gene orthologs were detected as commonly regulated in BP, CT and RM. They belong to Symbiosis (7 nodulation genes), Fatty acid and phospholipid metabolism (one gene encoding a putative fatty acid hydroxylase) and Chemotaxis and motility (*flg*G) functional classes (Table 3 and Additional file 1: Table S1). The induction of *nod* genes was expected since it has been widely described as a consequence of the perception of compatible flavonoid(s) by NodD transcriptional regulators [42]. The up-regulation of genes from the similar *nod* operons of BP (*nodBCIJHASUQT*, Fold Change (FC) 13–22×) and CT (*nodBCIJHASUQ*, FC 3.6–5.6×) was observed, as shown in Fig. 5. Their orthologs in RM were also up-regulated (FC 1.5 – 2×), but RM carries a different set of nodulation genes compared to BP and CT, with additional nodulation gene operons (*nodA1BCSUIJHPQ*, *nodM*, *nodO2*, and 3 *nodD* copies) of which some were regulated (Fig. 5). However, while BP and CT *nod* genes were induced and expressed at a relatively high level (FC of 5-22×) it was not the case for RM (FC of 1.3-2×).

Aimed at better exploring the regulation of *nod* gene expression by *Mimosa* root exudates, a kinetic analysis of their transcription was performed by qPCR for each rhizobium. The relative expression pattern was established for *nodB, nodD* and *nodA* (in the case of RM which contains multiple copies, *nodA1, nodA2*, *nodD1* and *nodD3* were tested, Additional file 1: Figure S5).

**Fig. 6:**
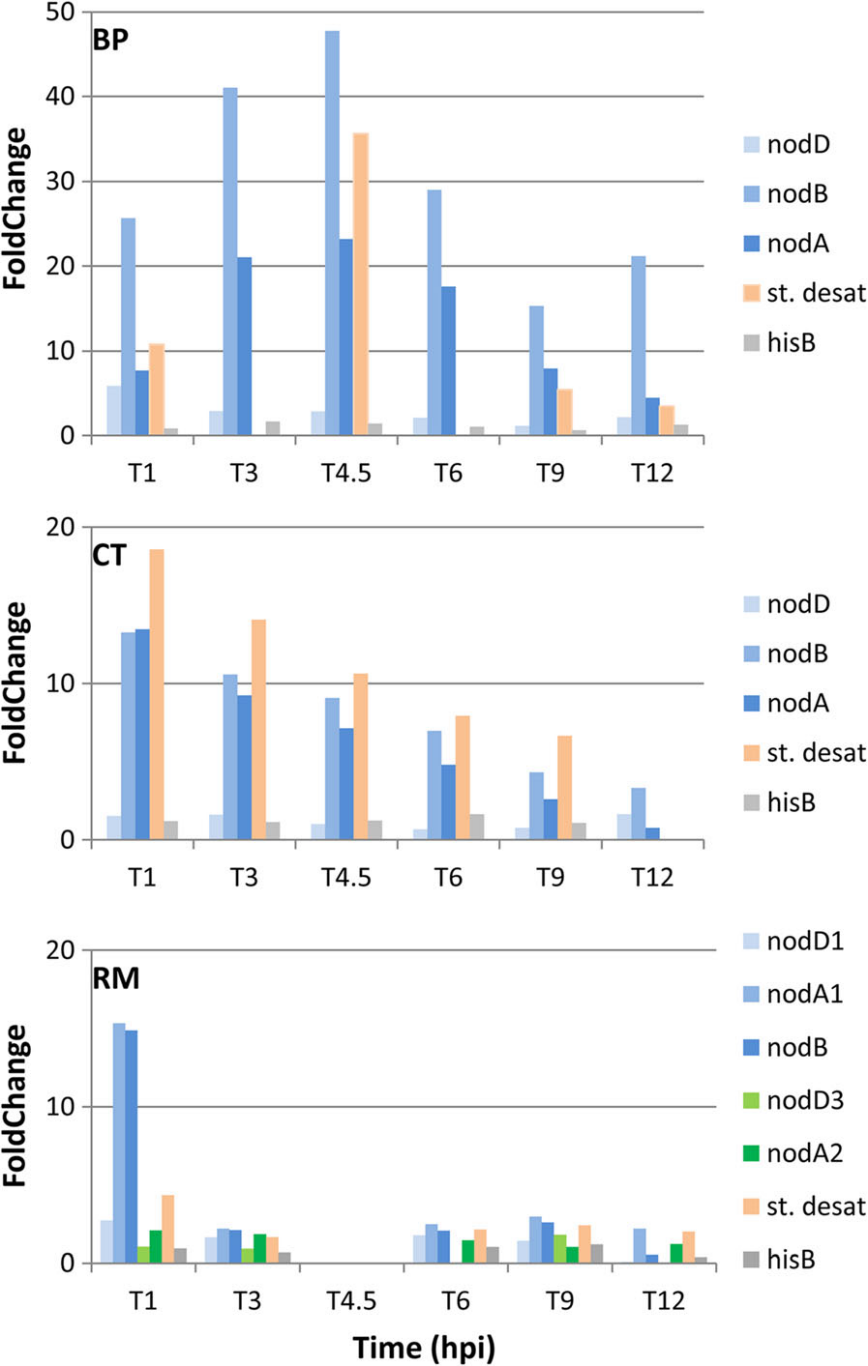
Kinetics of *nod* (*nodA*, *nodB* and *nodD)* and fatty acid hydroxylase gene expression as measured by RT-qPCR. The *uppS* gene was used to normalise data, and the other reference (*hisB*) gene expression profile is shown

As shown in Fig. 6, each rhizobium displayed different *nod* gene transcription kinetics in the presence of root exudates. In the case of BP, the maximum of *nodA* and *nodB* transcription was observed in the mid-exponential growth phase, at 4.5 hpi (FC 22× to 48×). CT and RM showed a maximum of *nodA(1)* and *nodB* up-regulation (13× and 15×, respectively) 1 h after induction. The signal decreased slowly in CT, but in RM it decreased rapidly to a 2.2× FC 2 h later. This result could explain the weak induction level obtained from RNA-seq data for RM *nod* genes performed at 6 hpi. No induction of *nodA2* (BN77v2_p2140039) was observed in parallel. To our knowledge these results show, for the first time, differences in symbiotic responses of rhizobia stimulated in the same way by root exudates. Clearly, both beta-rhizobia responded with important and persisting up-regulation of genes involved in Nod Factor biosynthesis and transport, contrary to RM. We can hypothesize that this difference could contribute to the competitive deficiency of RM in comparison to BP or CT as observed in [35]. Interestingly, a similar response was observed in the case of other alpha-rhizobia. When induced by bean root exudates, *Rhizobium tropici* strain PRF81 up-regulated the transcription of its *nod* genes 5 min post induction [47] followed by a decrease of the transcription signal within 15 min. *Sinorhizobium fredii* NGR234 also showed a rapid induction of *nod* genes, within 1 h post induction, although no information is available about the persistence of the induction level [48].

Another commonly regulated gene was related to the “Fatty acid and phospholipid metabolism” bioprocess. This gene (BURPHP2_0638, FC 27×; pRALTA_0471, FC 2.4× and BN77v2_p2140043, FC 1.74×) encodes an integral membrane protein containing a putative domain belonging to the “sterol desaturases and fatty acid hydroxylase superfamily”. The three orthologous genes share 80.3 and 78.7% of amino-acid identity (BP-CT and BP-RM, respectively), and less than 40% amino acid identity with orthologs from other bacterial genomes. Further studies are required to infer the function of these pSym-located putative “fatty acid hydroxylases” which showed several interesting features such as i) their up-regulation by root exudates with an expression pattern similar to *nod* genes as studied by qPCR (Fig. 6); ii) it is preceded by a *nod*-box sequence in CT (pRALTA_0471) [31], and we also detected a putative *nod*-box preceding each ortholog in BP (543894–543,880 bp in BURPHP2) and RM (251,926–251,950 bp in BN77v2_p2). This gene may thus represent a new specific nodulation gene for the symbiosis with *Mimosa pudica*.

The last commonly up-regulated gene in all three rhizobia is *flg*G (BURPHK1_3188, FC 1.7×; RALTA_B0282, FC 2.4× and RN77v1_1547, FC 2.1×) which is part of the flagellar components operon and encodes a protein of the basal-body rod.

Apart from the nine commonly regulated orthologous genes, we could observe common responses at the functional and biological process levels. Processes such as sensing, chemotaxis, flagella-driven motility and swarming, were all detected as regulated functions in our transcriptomic datasets. These functions are crucial for bacterial colonization of roots and have been found to be induced by root exudates in many rhizospheric bacteria [44–46]; differences in the responses to these functions may strongly influence the competitivity of bacterial symbionts [49]. We observed that the general chemotaxis and flagella-driven motility processes seemed to be regulated differently in the three bacteria. In RM we could observe the up-regulation of a whole region (RHI36v1_1529–1540) containing operons coding for chemotaxis (*che* and *mot*), flagella biosynthesis (*flg*, *fli*, *fla*, *flb*, *flh*) and flagellar motor components (*fliGNM* and *motA*). BP also showed a slight up-regulation of operons coding for chemotaxis (*che*) and flagella biosynthesis operons (*flg*, *fli*, *flh*) (Table 3, Additional file 1: Table S1). In CT, chemotaxis (*che*) and flagella biosynthesis operons (*flg*, *fli*, *flh*, *mot*) are regulated at a relatively low level, with an up-regulation for only two CDS (*flgA* and *flgG*).

Commonly regulated functions also comprised several histidine kinases, response regulators of two-component systems, methyltransferase of chemotaxis proteins, methyl-accepting chemotaxis proteins and putative chemoreceptors (Table 3, Additional file 1: Table S1). All these potential regulators could reflect differences between rhizobia in their capacity to sense/recognize and activate chemotaxis toward components of *M. pudica* root exudates.

We also observed that some regulated genes in the three rhizobia were linked to copper and zinc homeostasis in the presence of RE (Table 3). An up-regulation of metal efflux systems was illustrated in BP by the induction of the *cop* operon (BURPHK1_2204–2208, FC 2.15–4.05×) involved in copper resistance. In CT, many metal transporters were regulated: the *copSRABCD* operon (RALTA_B1970-B1965, FC 7.9-11×) coding for proteins involved in copper detoxification by extrusion of Cu2^+^ [50], genes of the *czcA2B2C2I2* operon (pRALTA_0640–0645, FC 1.61× and 1.54×) encoding components of an RND-HME efflux complex conferring resistance to Co, Zn and Cd [51], the *cupA2* gene (RALTA_A3123, FC 1.8×) encoding copper transporting P-type ATPase and its predicted partner *CupZ* (RALTA_A3123, FC 1.95×) encoding a copper ion binding metal transporter, and by a *zntA* gene encoding a cation (Cd)-efflux ATPase (RALTA_B1454, FC 2.16×). Concerning metal transport and/or homeostasis systems we observed in BP the down–regulation of the *cutC* operon (BURPHK1_0153–0154, FC − 2.0×) coding for copper homeostasis protein (BURPHK1_0154), and in CT the down-regulation of a gene encoding a divalent heavy-metal cations transporter (RALTA_A2241, FC − 3.85×). The induction of copper efflux-encoding genes has already been reported as an adaptation to the rhizosphere in alpha-rhizobia but also in other rhizospheric micro-organisms: *R. leguminosarum* bv. *viciae* showed an up-regulation of *copA*, *copC* (copper efflux) and *actP/cueA* (putative P-type ATPase) genes when induced with hesperetin and in the pea rhizosphere [49], while the *copSR* genes of *Pseudomonas syringae* [52] and the *cueA* gene (P1-type ATPase) of *P. fluorescens* SBW25 [53] were also up-regulated in the sugar beet rhizosphere.

### *Burkholderia* and *Cupriavidus* common responses: Beta-rhizobia

Despite the fact that *Burkholderia* and *Cupriavidus* belong to Betaproteobacteria, they are taxonomically distant, and in this study we detected only 51 commonly regulated orthologous genes between BP and CT. Apart from the nine genes already described as being commonly regulated in the three rhizobia, the other 42 genes represented 13 putative functional classes (Table 3, Additional file 1: Table S1). Among the up-regulated genes, eight genes belonging to the “Energy metabolism” and “Transport and binding” functional classes were associated with putative systems for the catabolism of RE components. One of the genes encodes a putative beta-ketoadipate enol-lactone hydrolase (BURPHK1_0214, FC 1.93× and RALTA_A0090, FC 1.57×) which catalyses a common step in the catabolism of protocatechuate and catechol by bacteria [54]. The importance of protocatechuate catabolism was demonstrated as an important capacity for the growth of *Rhizobium leguminosarum* bv *viciae* in the pea rhizosphere [55]. Two other genes, localized in orthologous operons, encode a putative hydroxymethylglutaryl-CoA lyase (BURPHK2_1264, FC 7.86× and RALTA_A1930, FC 2.12×) and a putative caiB/baiF family CoA-transferase (BURPHK2_1263, FC 8.95× and RALTA_A1931, FC 1.98×) involved in the mevalonate pathway for isoprenoid catabolism [56]. We also detected the up-regulation of a three gene operon encoding a sugar ABC transporter system (BURPHK2_2498–2503, FC 1.5–1.8× and RALTA_A2001–2006, FC 4.2–5.3×), a putative glycerol kinase (*glpK*, BURPHK1_0447 FC 2.58× and RALTA_A2010, FC 3.21×) and a putative sn-glycerol-3-phosphate dehydrogenase (*glpD*, BURPHK1_0448 FC 6× and RALTA_A2011, FC 3.54×). These genes show homologies to genes described in *R. leguminosarum* bv. *viciae* 3841 and involved in glycerol transport and catabolism, mutants of which are affected in their competition for nodulation [57].

Concerning the “amino acids transport and metabolism” bioprocess, two genes were up-regulated. The first one (*dadA*, BURPHK1_0823, FC 3.81× and RALTA_A0798, FC 5.04×) encodes a D-amino acid dehydrogenase involved in degradation of D-amino acids leading to the formation of corresponding oxoacids that can enter the Krebs citric acid cycle and glycolysis. The second one (BURPHK1_0327, FC 1.78× and RALTA_B0072, FC 1.65×) shares poor identity (25%) with LeuA (encoding a putative 2-isopropylmalate synthase) and may be involved in the metabolism of amino acids. In parallel, we observed a down-regulation of genes coding for branched-chain amino acid transporters, with two operons in BP (BURPHK1_0631–0635, FC − 2 to − 2.85× and BURPHK1_2430–2433, FC − 2.5 to -3×) and two operons in CT (RALTA_A1326–1331, FC − 2.6×; and RALTA_A3113–3117, FC − 2.5×). Branch-chained amino acids have been shown to be important for effective symbioses between BP and *Mimosa pudica* [58].

We also observed an adjustment of the central metabolism of beta-rhizobia. First with the up-regulation of the *aceA* gene (BURPHK1_1476, FC 1.64× and RALTA_A1766, FC 11×) encoding an isocitrate lyase, an enzyme of the glyoxylate bypass of the tricarboxylic acid (TCA) cycle. The FC was much higher in CT, which is linked with the up-regulation of two other genes of the glyoxylate bypass in CT (see CT specific response section). Rhizobia use the TCA cycle for the oxidation of organic acids [49], a feature that could be also present in beta-rhizobia. Further, we observed a down-regulation of an operon of eight genes coding for subunits of a NADH-quinone oxidoreductase (*nuo* operon: BURPHK1_2160–2173, FC-1.8 to − 2.2× and RALTA_A1034–1047, FC − 1.5 to − 1.6×), involved in the generation of the proton force which can be used for the synthesis of ATP, the active transport of various nutrients and the ATP-dependent rotation of the flagella [59].

Bacterial survival in the rhizosphere, apart from adaptation toward nutrient sources, also involves dealing with environmental challenges caused by the presence of oxidative stress and toxic compounds. In our study, both beta-rhizobia showed a common response toward a putative oxidative stress by up-regulating genes coding for antioxidant-proteins classified in the “Detoxification” functional group. These were genes coding for a peroxiredoxin-like protein (BURPHP1_1192, FC 1.62× and RALTA_B2016, 1.58×), a putative thioredoxin reductase (BURPHK2_0718, FC 4.57× and RALTA_A1176, FC 2.1×) and a glutathione S-transferase (BURPHK1_0157, FC 1.86× and RALTA_A1551, FC 1.54×). Induced transcription was also observed in BP for an *ahpD*-like gene (BURPHP1_1209, FC 5.37×) coding for a putative antioxidant alkyl hydroperoxide reductase, and in CT for two putative universal stress proteins of the *uspA*-family (RALTA_A0490, FC 1.81× and RALTA_A2972, FC 1.58×). The up-regulation of genes encoding putative peroxiredoxin, thioredoxin, glutathione S-transferase and UspA, was previously observed in different plant-microbe early interactions as *R. leguminosarum* bv. *viciae* in the pea rhizosphere [49]. Surprisingly we did not observe this type of response in our alpha-rhizobium (RM) but only in the beta-rhizobia. Concerning other sensing and detoxification systems we detected the up-regulation of a NO dioxygenase (BURPHK2_1027, FC 1.83× and RALTA_A2986, FC 2.8×) which converts NO to N_2_O or to NO_3_^−^ [60], involved in nitric oxide (NO) sensing and detoxification. NO has been shown to be an important factor for the establishment of *Sinorhizobium*-*Medicago* symbiosis [61].

Finally, both beta-rhizobia showed similar up-regulation of several operons encoding efflux systems. These were in BP, two systems belonging to resistance/nodulation/cell division (RND) family and of unknown function BURPHK1_2604–2606 (FC 2.2–5.2×) and BURPHP2_0566–0568 (FC 4.8–7.3×), the second being located up-stream of the *nod* operon. In CT three RND systems were up-regulated: a multidrug efflux system *mdtABCD* (RALTA_A0537–0540, FC 1.73–1.47×), a multidrug transporter (RALTA_A3171–3173, FC 3.69–2.31×) and a putative efflux pump similar to acriflavine/multidrug efflux *acrAB* and *nodT* (RALTA_B0936–38, FC 1.5×). Indeed, it has been found that the RND systems might have a relevant role in the interaction of bacteria with their plant hosts, from the first steps of colonization [62] to their survival in plant tissues [63]. It was also demonstrated in alpha-rhizobia that specific RND systems may affect the number of nodules formed on host roots [64], the level of nitrogen fixation in nodules [65] and the rhizosphere competitiveness of rhizobia [49]. Finally, we observed the up-regulation of four genes coding for proteins involved in regulatory mechanisms including a sigma 54 factor, and regulators of the MerR, GntR and LuxR families (Additional file 1: Table S1).

### *Burkholderia* and *Rhizobium* common responses

Twenty eight orthologous genes were found to be commonly regulated in BP and RM. Among them, 19 were up-regulated (including *nod* genes) and nine were down-regulated. They were classified into eight functional groups (Additional file 1: Table S1). Among the up-regulated genes belonging to the “Energy metabolism” bioprocess, we observed induction of two systems. The first one is represented by the genes *iolB*, *iolC* and *mmsA*/*iolA* (Additional file 1: Figure S8). In RM these genes exhibit a high nucleotide identity (from 82.8 to 94.8%) with orthologs in *R. leguminosarum* and *S. meliloti* encoding a *myo*-inositol catabolism system [66]. The capacity of both bacteria to use myo-inositol as a single carbon source was observed on API GN20 galerie tests (Additional file 1: Figure S9), while CT was unable to. The presence of inositol and pinitol (3-O-Methyl-D-chiro-inositol) was detected in *M. pudica* RE, the last one representing the most concentrated component of RE (Table 4). These genes could thus be involved in catabolism of inositol (and derivative compounds), a key pathway involved in host nodulation competitiveness in *S. meliloti* [67]. The second “Energy metabolism” bioprocess is represented by four genes (BURPHK2_0654–0657, FC 1.84–2.32× and BN77v1_3275–3278, FC 2.08–2.32×) encoding a phosphate acetyltransferase (Pta), an acetate kinase (AckA) and an enoyl-[acyl-carrier-protein] reductase (FabI). It was described in *S. meliloti* as being induced by phosphate starvation [68].

**Table 4:** Composition of *M. pudica* root exudates and its final concentration when added into culture medium for rhizobium induction

Components	Concentration (μM)	% error
glucose	0.49	7.6
fructose	**109.72**	12.6
ribose	1.10	2.4
xylose	0.25	6.4
alanine	0.27	4.4
isoleucine	0.17	11.8
leucine	0.17	11.9
serine	0.18	10.5
aspartic acid	0.90	2.6
gluconic acid	**114.24**	2.7
glutamic acid	1.53	4.53
malic acid	2.76	2.3
pinitol	**145.14**	0.5
inositol	0.73	3.1
polyphenols^a^	10–15	nd

^a^ polyphenolic compounds measured as equivalent of ferulic acid (μg.ml^−1^). Major compounds concentrations are indicated in bold

We also observed a down-regulation of four genes belonging to the “Transport and binding proteins” bioprocess, encoding putrescine/spermidine ABC transport systems (BURPHP1_0401–0404, FC -2× and BN77v1_3961–3964, FC − 1.75 to − 5.5×). γ-aminobutyric acid (GABA) and putrescine were shown to be present in root exudates of numerous plants and they seem to play a role in nitrogen cycling in both the rhizosphere and within nodules [69].

### *Cupriavidus* and *Rhizobium* common responses

Apart from the up-regulation of the nodulation gene operon and a fatty acid hydroxylase gene, there was only three orthologous DEG in CT and RM, but they are regulated in opposite directions, including *cobU* and *fliI*, and a gene encoding a protein of unknown function. Such absence of overlap in the responses of both symbionts to root exudates mirrors their different adaptation to symbiosis.

### *Burkholderia phymatum* specific responses to RE

For specific responses of each organism, we compiled both RE-regulated genes specific to each rhizobium and those only regulated in one bacterium but with orthologs in the other rhizobia. Here we present the most interesting, in our opinion, responses of BP, CT and RM, in the context of plant-bacterial interactions. The complete list of specific regulated genes of the three rhizobia is presented in Additional file 1: Table S1.

The BP-specific response is represented by 755 genes with assigned function (288 genes up-regulated and 467 genes down regulated, within 27 assigned bioprocess functions) and 557 genes of unknown function.

BP activated the transcription of several genes encoding functions known to be involved in the interaction between bacteria and plants (Table 3): the *acdS* gene encoding 1-aminocyclopropane-1-carboxylate (ACC) deaminase (BURPHP2_0697, FC 2.24×) and a putative rhizobitoxine biosynthesis operon of seven genes (BURPHP2_0646–0653, FC 2.4–3.4×) (Additional file 1: Figure S10). The first four genes of this operon show high homology (from 52 to 70% of nucleotide identity) to the *rtxACD* genes involved in the biosynthesis of rhizobitoxine in *Bradyrhizobium elkanii* USDA 76 [70]. The fact that RtxACD were shown to be essential and sufficient for the biosynthesis of rhizobitoxine [71] suggests that BP could be able to synthetize rhizobitoxine. Both ACC deaminase and rhizobitoxine were shown to play a positive role in establishing symbiosis between rhizobia and their legume hosts by enhancing the nodulation process: rhizobitoxine by inhibiting the activity of the plant ACC synthase involved in ethylene biosynthesis in host roots [72] and ACC deaminase by degradation of ACC, the immediate precursor of ethylene [73]. We also detected the up-regulation of an operon (BURPHP2_0632–631, FC 1.7–1.8×) encoding two putative proteins involved in the biosynthesis of the plant hormone indole-3-acetic acid (IAA). The role of IAA biosynthesis by rhizobia during symbiosis remains unclear. It was demonstrated that in *Sinorhizobium sp*. NGR234 flavonoids induce IAA production via the transcriptional regulators NodD1 and NodD2 [74], and a strain of *R. leguminosarum* bv. *viciae* (strain RD20) overproducing IAA elicits the development of fewer but bigger nodules on vetch, and with an enlarged and more active meristem, compared to nodules infected by the wild-type strain [75]. Another highly up-regulated gene possibly involved in the plant-bacterial interaction is BURPHP2_0639 (FC 19×), which encodes a putative endoglucanase. This gene is located on the pSym, down-stream of the earlier described putative fatty acid hydroxylase (BURPHP2_0638, FC 27×), and represent the fifth most induced gene in BP. It shows 27% amino acid identity to CelB, an endoglucanase of the phytopathogenic *Pectobacterium carotovorum* LY34 strain and involved in plant cell wall degradation [76]. The only rhizobial cell-wall-degrading enzyme described to be involved in early symbiotic interaction was CelC2 of *R. leguminosarum* bv. *trifolii* [77, 78]. Even if our BP endoglucanase shows no homology to CelC2, its strong induction by RE may, however, be a good indicator of its implications for early symbiotic events and makes it a good candidate for further study. We detected orthologs of these genes in the genomes of CT or RM: *acdS* is present in the RM genome (BN77v2_p270023, FC 1×) and the endoglucanase encoding gene in the CT genome (pRALTA_0250, FC 1×). It is noteworthy that the three above mentioned mechanisms are encoded by genes localised on the pSym. Several nitrogen fixation-related genes were also highly induced in BP: an operon containing conserved fragments of *nifZ* (BURPHP2_0643, FC 16×) and *nifB* (BURPHP2_0640, FC 11×), as well as the *fixU* gene (BURPHP2_0644, FC 11×), and several other *nif* and *fix* genes (BURPHP2_0596–0614, FC 1.6–2.2×) (Table 3).

BP specific responses which could contribute to symbiont-host plant interactions concerned the differential expression of secretion systems (Table 3). BP responded to RE by up-regulation of a T4SS (BURPHP2_0345–367, FC 1.47×–4.34) and a T6SS (T6SS-2, BURPHP1_0625–645, FC 1.53×-1.93×), and by down-regulation of another T6SS (T6SS1, BURPHP1_0486–506, FC − 2.0× to − 2.7×). The up-regulated T6SS consist of genes arranged into two modules transcribed in opposite directions (Additional file 1: Figure S11). This T6SS was classified as T6SS-2 on the basis of neighbour-joining phylogenetic analyses of VgrG, Hcp, ClpV, IcmF, and the lysozyme-like protein VC_A0109 [79]. The closest (but incomplete) locus synteny was found with the endophyte *B. phytofirmans* PsJN [80], the animal pathogen *B. mallei* ATCC 23344 [81], the causative agent of melioidosis *B. pseudomallei* K96243 [82], the rice sheath pathogen *B. gladioli* BSR3 [83] and with the mango plant pathogen *Burkholderia* sp. TJI49 [84] (Additional file 1: Figure S11A). The first module of this T6SS-2 is composed of the *sciHIKN* genes, while the second module comprises a gene encoding a putative conserved protein, the *vgr* gene and a gene encoding a putative chitinase. Hcp (SciK) and Vgr are the essential components of T6SS and current models of T6SS suggest that Hcp and Vgr form a tubular structure of stacked Hcp hexamers terminated by a VgrG trimer, that functions as a perforating device toward the targeted cells [85]. The Vgr protein (encoded by BURPHP1_0643) possesses a lysozyme domain in C-terminal (as predicted by InterProScan). Downstream of the *vgr* gene another up-regulated gene is present encoding a putative enzyme harbouring muramidase (504–742 aa) and peptidoglycan hydrolase (33–164 aa) catalytic domains. T6SS have been described as important factors in interbacterial species competition for niche occupancy, by causing damage to competitors by perforation [86, 87]. The induction of such T6SS-2 could be an important factor in the high competitivity of BP against its rhizobial competitors [35, 88]. This is, to our knowledge, the first report of the induction by RE of genes encoding a T6SS in rhizobia.

The second BP T6SS (BURPHP1_0486–506), which is down-regulated by RE, shares high identity and synteny with T6SS in *Burkholderia* symbionts of *Mimosa* (*Burkholderia* sp. CCGE1002, JYP251), plant endophytes (*B. kururiensis* M130) or soil bacteria (*B. terrae* BS001) (Additional file 1: Figure S11B). Rhizobial T6SS (as T4SS) can have positive or negative effects on host nodulation by suppressing plant immune systems [89]. Although the direct influence of T4SS on symbiosis was demonstrated by mutagenesis in alpha-rhizobia [90, 91], there is only one report of the role of a T6SS in nodulation in *R. leguminosarum* where the T6SS prevented nodulation on *Pisum sativum* cv. Rondo [92].

The BP T4SS consist of the Mpf (Mating pair formation) genes *virB1* to *virB11*, as well as the regulator *virD4*, and can be classified as of the T4ASS type based on its homology to the complex VirB/D4 of the plant pathogen *Agrobacterium tumefaciens* [93] (Additional file 1: Figure S12). All genes of this T4SS are moderately up-regulated (1.47 x – 4.34 x FC), as does two genes located up-stream (BURPHP2_0369–0370, 2× and 3.5× FC) which code for a putative transcriptional regulator (XRE family) and a putative sensor protein with a PAS domain which could be involved in the T4SS regulation. Four other up-regulated genes (BURPHP2_0345–348, 2.2 x – 2.5 x FC) located down-stream of the T4SS encode a putative Dtr (DNA transfer and replication) module [94] of the conjugative machinery. The presence on the pSym and simultaneous up-regulation of these conserved Dtr and Mpf modules suggest the involvement of the BP T4SS in the conjugative transfer of the pSym.

We also detected a down-regulation of genes encoding putative proteins of the general secretion pathway (Sec-pathway) classified also as Type 2 Secretion System (T2SS) by which proteins are transported across or inserted into the cytoplasmic membrane [95]. These genes are *secY* (BURPHK1_3037, FC − 2.4×), *secE* (BURPHK1_3073, FC − 1.56×) and *secDF* (BURPHK1_2730–2731, FC − 1.5×). A slight decrease of expression was also observed for the genes *secA* (BURPHK1_2871, FC − 1.3×) and *secB* (BURPHK1_0294, FC − 1.3×). The Sec translocation system is involved in the secretion of the vast majority of secreted proteins, and it was also shown to be involved in the secretion of toxins in *Listeria monocytogenes* and *Bacillus cereus* [96, 97]*.*

Other specific responses of BP concerned the bacterial catabolism and metabolism of aromatic compounds, polyamines and amino acids (Table 3). Our data showed the up-regulation of genes coding for enzymes possibly involved in the catabolism of aromatic compounds: a putative 4-oxalocrotonate tautomerase (BURPHK2_0305, FC 1.74×) involved in the degradation of catechol, a putative mandelate racemase/muconate lactonizing-like protein (BURPHP1_0070, FC 1.95×) involved in aromatic acid catabolism, a dioxygenase annotated as a putative glyoxalase/bleomycin resistance enzyme (BURPHK2_1088, FC 1.65×), a DOPA 4,5-dioxygenase (BURPHK2_2585, FC 2×) involved in aromatic ring-cleaving, and a dehydratase (BURPHK1_1833, FC 1.5×) which could be involved in the degradation of sistosterol-like molecules or fatty acids. Concerning polyamines, several putrescine transporters were down-regulated (see BP & RM common responses) and several genes encoding putative enzymes involved in the degradation of γ-aminobutyric acid/putrescine (*puuC*, BURPHK2_0522, FC 1.8×; *puuDA*, BURPHK2_0525–0526, FC 1.4–1.7×; *puuC*, BURPHK1_2348, FC 1.55×; BURPHP1_1138–1140, FC 2–2.5×) were up-regulated. Another observation concerns the down-regulation of 17 genes encoding enzymes involved in amino acid (leucine/valine, histidine, phenylalanine/tryptophan, lysine, arginine, tyrosine and glutamate) biosynthesis, as well as cysteine, histidine and methionine transporters (Table 3). The down-regulation of different types of amino acid and of polyamine transporters suggests a reorganization of the metabolism of BP to the presence of RE compounds.

Finally, we detected the up-regulation of genes coding for a putative siderophore non-ribosomal peptide synthase (BURPHK2_1036–1049, FC 1.54–2.34×) known to play a pivotal role in iron access competition. The BraI/BraR quorum sensing regulation system (BURPHK2_1465–1467) [98] and Contact-Dependent growth Inhibition Systems (CDI) CdiAB [99] (BURPHP2_0083, 0089, 0113, 0130) were not regulated in our RE-induced conditions.

### *Cupriavidus taiwanensis* specific response

The CT specific response is represented by 186 genes with assigned function (104 genes up-regulated and 82 genes down regulated) and 134 genes of unknown function. The most pronounced CT specific response in terms of number of genes (up- and down-regulated) concerns the bioprocesses “Energy metabolism”, “Transport and binding”, “Regulatory functions” and “Excrete” (Fig. 4).

An up-regulation of genes coding for proteins involved in root attachment was detected in CT: the operons *pilVWXYE* coding for the biogenesis of the type 4 fimbriae (twitching motility that can be important factors in host colonization [100]) (RALTA_A2329–2324, FC 1.4–2.08×; and RALTA_A2331–2335, FC 0.9–1.9×) and *pilQPONM* coding for the assembly of the type 4 fimbriae (RALTA_A2895–2899, FC 1.53–1.94×), and a monocistronic gene coding for a PilX-related protein (RALTA_A0505, FC 1.95×). PilX was shown to be required for surface and endophytic colonization of rice as well as twitching motility of *Azoarcus* sp. BH72 [46], and required for attachment and invasion of human cells by the pathogens *Pseudomonas aeruginosa* and *Neisseria meningitidis* [101, 102].

Two putative chromosomal toxin/antitoxin systems (TA) were up-regulated: *kluBA* (RALTA_A0064–0065) for which only the antitoxin was induced (RALTA_A0065, FC 1.52×), and RALTA_B2161–2162 for which both toxin and antitoxin were induced at a relatively high level (FC 2.13–2.24×). This latter system shows synteny and 41.3% identity with a TA system localized on the pSym (pRALTA_0622–0623, FC 1.43–1.48×). The physiological function of chromosomal TA systems is still controversial. The activation of TA under various stress conditions suggests a function in general stress management and of special importance during the adaptation of rhizosphere bacteria to oligotrophic conditions as well as a role in plant-bacterial interactions [103, 104]. No up-regulation of genes encoding putative toxin/antitoxin systems was observed in BP or RM.

CT up-regulated several genes involved in the glyoxylate bypass pathway: an isocitrate lyase (*aceA1*, RALTA_A1766, FC 11×) and its paralog (*aceA2*, RALTA_B0267, FC 1.89×) and a malate synthase (*aceB*, RALTA_A1760, FC 1.6×). Induction of these genes was also observed for *R. leguminosarum* in its specific host rhizosphere (pea) and suggests the catabolism of short chain (C2) organic acids [49]. In CT the glyoxylate bypass could also be linked with PHB degradation that would provide more acetyl-CoA [105]. CT up-regulated two out of seven genes encoding intracellular poly(3-hydroxybutyrate) depolymerases (*phaZ3* RALTA_B1788, FC 1.54× and *phaZ4* RALTA_B2163 FC 1.52×) as well as one gene encoding a beta-hydroxybutyrate dehydrogenase (RALTA_A1256, FC 1.35×). At the same time, CT down-regulated an acetoacetyl-CoA reductase (*phaB2*, RALTA_A1758, FC − 2.44×) and phasin (*phaP2*, RALTA_A1759, FC -2×) involved in PHB synthesis. Studies of [106, 107] on the plant pathogens *Rhodococcus fascinans* and *Xanthomonas campestris* have suggested an important role of the glyoxylate cycle in plant-microbe interactions, as malate synthase-deficient mutants were impaired in their pathogenic capacities. However, the glyoxylate cycle does not seem to be important during symbiotic interactions in alpha-rhizobia, as isocitrate lyase null mutants of *R. leguminosarum* and *S. meliloti*, and malate synthase null mutants of *R. tropici* and *S. meliloti* were unaffected in their nodulation capacities [105, 108, 109]. PHB synthesis and degradation is of great importance for rhizobial persistence and survival during starvation [110]. Concerning the glyoxylate cycle and PHB degradation, our results showed an interesting difference between CT and the other two rhizobia, BP and RM.

CT up-regulated two operons involved in nitric oxide (NO) sensing and detoxification: a NO-sensing transcription repressor (*nsrR*, FC 6.3×) and a NO dioxygenase (*hmp*, FC 2.8×) which converts NO to N_2_O or to nitrate (NO_3_^−^) (RALTA_A2987–2986) [60] and a NO-binding di-iron protein (*norA*, FC 6.3×) that modulates the NO-responsive expression of the *norAB* operon by lowering the free cytoplasmic concentration of NO [111] (RALTA_B2089–2087). The up-regulation of two independent NO detoxification systems suggests the synthesis of NO in the CT cells. CT nitrate and nitrite reductases (*napCBA1DE* and *nirSCFDGHJNE*, respectively) are not up-regulated and seem to be expressed at a relatively low level, and no gene(s) coding for NO synthase could be identified. Concerning CT *nif* and *fix* genes, these are not regulated by RE but are transcribed at a high level (with *nifHDK* FC at 1×, 25,000 to 29,000 reads per CDS), contrary to BP and RM (which carry two nitrogenase encoding loci) in which *nif* and *fix* genes are transcribed at a very low level under both conditions. NO is not known to be a nitrogen fixation by-product, but its presence in nodules is known to inhibit nitrogenase activity [112], so such detoxification systems in CT could be linked to symbiosis. Multidrug efflux systems were also up-regulated in CT, such as *mdtABC* (RALTA_A0538–0540, FC 1.7×).

CT possesses a specific T3SS (RALTA_B1250–1262) which is not induced by RE and is not expressed (less than 10 reads for each CDS). It was previously shown that its inactivation had no effect on *Mimosa pudica* nodulation but allowed CT to establish chronic infections and to fix nitrogen in *Leucaena leucocephala* [113], which is in line with our RNAseq results showing neither induction nor expression of this T3SS under our experimental conditions.

### *Rhizobium mesoamericanum* specific responses

The RM specific response to root exudates is represented by 117 genes with assigned function (97 genes up-regulated and 20 genes down regulated, with 17 assigned functions) and 69 genes of unknown function (Additional file 1: Table S1). The most pronounced RM-specific response in terms of number of genes involved (up- and down-regulated) concerns the “Chemotaxis and motility” (Biop 15.2), “Transport and binding” (Biop 7) and “Cell envelope” bioprocess (Biop 14) (Fig. 4).

The first interesting feature in the RM-specific response is the induction of specific nodulation genes absent from BP and CT, namely *nodM*, *nodO1* and *nodO2* (BN77v2_p2180030 FC 2.4×, BN77v2_p290009 FC 1.44×, BN77v2_p2180035 FC 1.83×, respectively) (Fig. 5). The *nodM* gene encodes a fructose-6-phosphate glutamine aminotransferase and provides glucosamine-6P that are assembled by NodC to form the Nod factor backbone [114, 115]. It is a paralog of the conserved bacterial gene *glmS.* BP and CT only carry the conserved *glmS* gene. The *nodO1* and *nodO2* genes encode calcium-binding proteins. NodO has been shown to insert into liposomes and form cation-selective channels in lipid bilayers and could, therefore, enhance the calcium spiking that is observed in root hairs upon Nod factor binding [116]. In *R. leguminosarum* bv. *viciae*, a type I secrection system (PrsDE) allowed the secretion of the NodO protein [117]. Interestingly, orthologs of PrsDE (66 to 69% amino acid identity) located on the RM pSym were also up-regulated by RE (BN77v2_p2120002-p2120004, FC 1.6–2.4×). RM also harbors two copies of the *nodA* gene, of which only one (*nodA1*, BN77v2_p280014, FC 1.9), is induced by RE (nodA2 is BN77v2_p2140039, FC 1.05) (Fig. 5).

Concerning chemotaxis and motility, RM up-regulated a gene encoding a chemoreceptor (*mcpE*, BN77v1_1522) upstream of a whole chemotaxis up-regulated operon *cheXYAWRBD* (BN77v1_1522–1530, FC 1.5–1.8×) and close to another up-regulated *motBCD* operon (BN77v1_1559–1563, FC 1.7–1.8). Other methyl-accepting chemotaxis proteins were up-regulated (*mcpA*, BN77v1_1799 FC 1.8×, BN77v1_1594 FC 1.6, BN77v1_1773 FC 1.6), and one was down-regulated (*mcpY*, BN77v1_p10178, FC − 1.7×). Rhizobia mediate chemotaxis by sensing molecules from root exudates using chemoreceptors, a phenomenon that allows the bacterium to activate several functions (including motility) to reach its host, and is important for host nodulation competitiveness [118]. We also observed the up-regulation of 31 motility genes (BN77v1_1532–1576, FC 1.5–2) localized downstream of the chemotaxis genes previously described, and involved in flagellar biosynthesis (motor switch *flhB*, *fliFGNM*, *motA*, basal body *flgBC, fliE, flgGAIH*, flagellin biosynthesis *flaABC*, flagellar hook *flgEL*). The up-regulation of these motility genes is in parallel with the downregulation of the *csgAB* genes encoding curlin biosynthesis enzymes involved in biofilm formation (BN77v1_p11016–11,018, FC − 2.6 to -3×), reflecting a switch of the bacterium to a mobile state.

Another specific response of RM concerns the “Cell envelope” bioprocess, by regulating genes involved in modifications of EPS and LPS composition. We detected the up-regulation of 18 genes encoding putative proteins involved in the biosynthesis of EPSII (BN77v2_p11446–11,463, *wgeBCDEFG*/*wgeA*/*wgdAB*/*expG*/*wgcA*/*wgaABDEF*, FC 1.9×) and of a gene encoding a negative regulator of EPS, PsiB (BN77v2_p2140032, FC 1.8×) [119], while in parallel 15 genes involved in EPSI were downregulated (*exsH, exoYF, aceC, pssP,* BN77v2_p10959–10,972, FC − 1.5 to -2×), indicating a shift from EPSI to EPSII of the RM EPS composition. We also observed the up-regulation of the operon *rfbABCD* (BN77v1_p11464–11,467, FC 1.7–1.8×) encoding proteins involved in *O*-antigen building, and which blocks biosynthesis of a putative galactopyranose mutase (*glf*, BN77v1_p10763, FC 1.9×), a putative rhamnosyl O-methyltransferase (BN77v1_0913, FC 1.78×) and a putative glycosyl transferase (BN77v1_2672, FC 2.1×). Exopolysaccharides have been shown to play different roles in symbiosis, especially for bacterial propagation in infection threads and suppression of host defenses [120, 121].

Several genes involved in sugar transport and/or catabolism, were up-regulated in RM: a fructose uptake ABC transporter (*frcKABC*, BN77v1_1335–13, FC 4–4.7×; fructose being the third main component of root exudates), a xylose epimerase (BN77v1_p11238, FC 4×) and an ABC transporter (BN77v1_p10666–10,668, FC 2.4–2.9×). We also detected the up-regulation of three genes (BN77v1_0504–0506, FC 1.53–2.11×) with high homology with the *mocDEF* (59.7 to 79.3% amino acid identity) from *S. meliloti* L5–30 and *R. leguminosarum* bv *viciae*, species in which these genes are part of the *mocCABRDEF* operon encoding a rhizopine catabolism pathway [122, 123]. Close to *mocDEF* was found an homolog of *mocB* (encoding the rhizopine-binding protein) within an ABC transporter operon (annotated as a galactoside transporter), which was also up-regulated by RE (BN77v1_0783–0785, FC 4–4.7×). The rhizopine catabolic capacity of *R. leguminosarum* also requires the presence of the *myo*-inositol degrading enzymes IolD (acetolactate synthase) and IolA (methylmalonate-semialdehyde dehydrogenase) [124]. We also observed in RM an up-regulation of genes involved in *myo*-inositol degradation (described in the “*Burkholderia* and *Rhizobium* common responses” section) and of the *idhA* gene (BN77v1_3490, FC 2.4×) encoding a putative Idh/MocA family oxidoreductase. Taken together, these data suggest that RM could catabolize rhizopine, an ability that has been shown to provide an advantage for competitive host nodulation in *R. leguminosarum* and *S. meliloti* [67, 125]. We also detected the up-regulation of an operon encoding a lipid acid hydrolase harboring a patatin-like domain and a membrane fusion protein (BN77v1_p10807–10,810, FC 2.4–3.4×). Finally, RM up-regulates genes involved in the formation of mechanosensitive channels (BN77v1_p10999, FC 1.9×; BN77v1_3475 FC 1.9×) which have been suggested to play a role in the response to hypo-osmotic shock in the rhizosphere [126].

## Conclusions

**Fig. 7:**
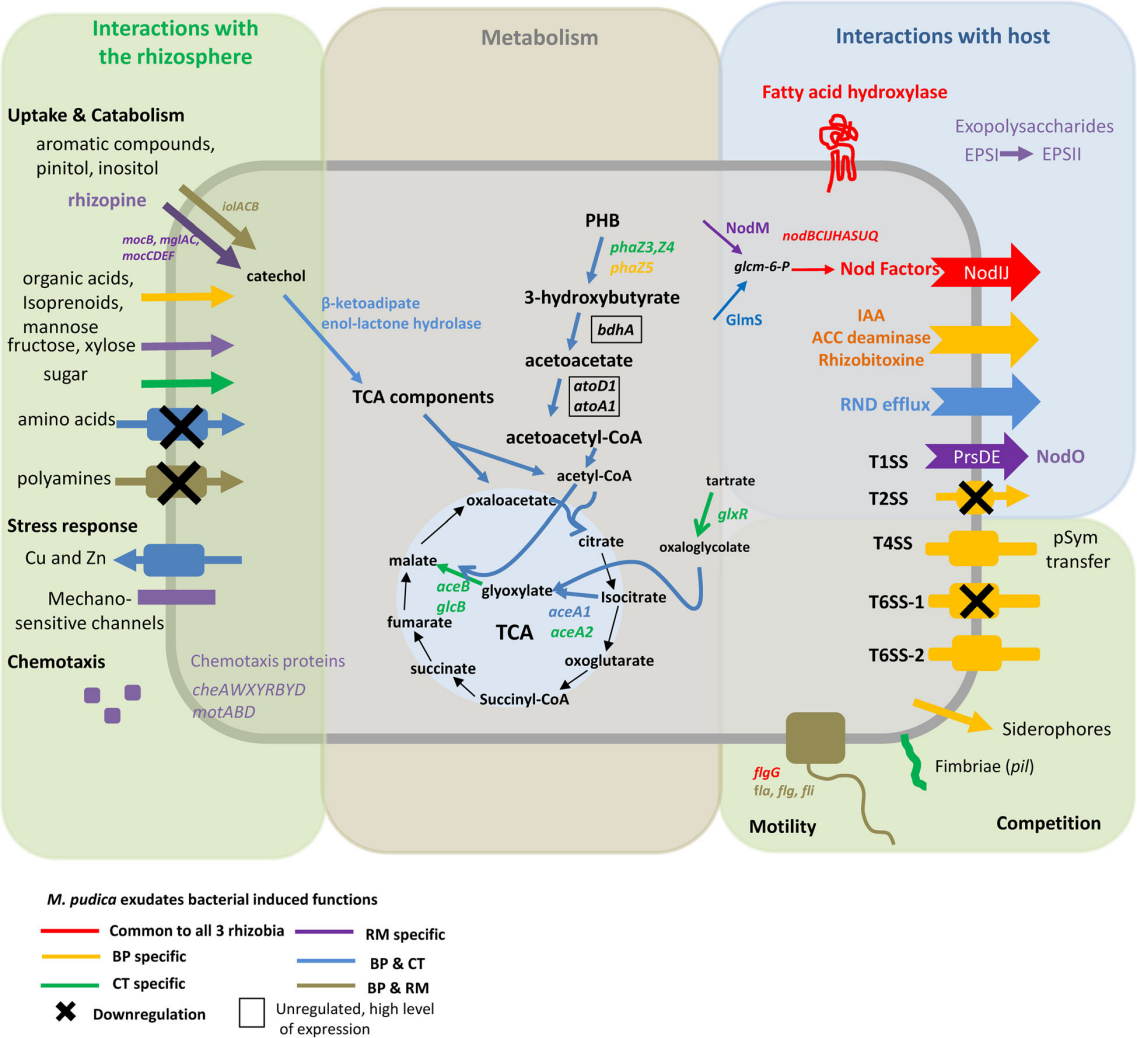
Illustration of the main functions regulated in BP, CT and RM in the presence of root exudates from *Mimosa pudica*. Functions commonly up-regulated among the 3 rhizobia, or specific, are colored differently (red: in all 3 rhizobia, orange: BP specific, green: CT specific, blue: BP & CT, purple: RM specific, brown: BP&RM. The cross indicates down-regulated functions

In this study we compared the transcriptomes of three different rhizobial symbionts of *Mimosa pudica,* in response to root exudates (RE) at mid-log exponential stage of growth. The main results are illustrated in Fig. 7. We observed that BP has the most substantial response to RE both quantitatively and qualitatively compared to CT and RM, which is in line with its high competitiveness recorded in previous work on *Mimosa* [29] and more recently on papilionoid legumes [88]. BP harbors and up-regulates in the presence of RE many genes known to be involved in plant-bacterial interactions, present in Alpha and Betaproteobacteria, including ACC deaminase, rhizobitoxin production, IAA biosynthesis, and a T6SS to probably eliminate its competitors. Such high transcriptomic reorganization to the sensing of RE, and the presence of many (almost all known) genes involved in plant growth promotion in BP, mirrors the long history of symbiosis between burkholderias and *Mimosa* species as inferred by phylogenetic analyses [30]. Such features were not observed in CT, which nodulates *Mimosa pudica* mainly in areas where it is invasive and not native, and where its main competitors (i.e. burkholderias) are counter-selected by environmental conditions (such as high soil pH or the presence of heavy metals) [5]. CT displays a “minimal” transcriptomic response to RE, reflecting a shorter history of symbiosis with *Mimosa pudica* [5], and/or the fact that it encounters its host under harsh conditions counter-selecting *Burkholderia* or *Rhizobium* spp., thus lowering the selective pressure for genes involved in competitiveness. Indeed, apart from nodulation genes, CT regulates only trophic or stress-related functions (e.g. heavy-metal transporters) when its competitors up-regulate many plant-related or competitiveness genes. Such a minimal response of CT towards symbiosis is correlated with its small symbiotic gene content, which was taken as an advantage for the transfer of symbiosis to its pathogenic relative, *Ralstonia solanacearum* [127, 128].

The only common response of the three rhizobia to RE is the up-regulation of the canonical nodulation genes, plus an additional fatty acid hydroxylase which is currently under investigation to prove its role in the symbiosis of *Mimosa pudica.* RM seems to up-regulate its nodulation gene content differently, with an earlier transcriptional response compared to BP and CT, and a decrease of expression after 1 h. RM also carries and up-regulates extra *nod* genes (*nodM*, *nodO1*, *nodO2*), illustrating a different history of nod gene acquisition and recruitment to symbiosis [10, 129]. RM also has many genes already described in alpha-rhizobia (such as in *R. leguminosarum* and *S. meliloti*) known to be playing a role in nodulation and/or competition in the rhizosphere (rhizopine catabolism, biofilm formation, motility …).

However, given we monitored one time point by RNAseq while kinetics by qPCR on *nod* genes revealed different induction profiles, we cannot exclude that these conclusions drawn from the RNAseq profiling might be different at earlier or later time-points of induction. In future work we will compare these alpha and beta-rhizobia at later steps of the symbiosis process to decipher the symbiotic specificities in both types of symbionts.

## Methods

### *Mimosa pudica* Root exudate (RE) preparation and composition

*Mimosa pudica* seeds (B&T world seeds; mix 50/50 var. *hispida* and *unijuga*) were surface-sterilized and germinated as described in [10]. For root exudation, *M. pudica* were grown in 48-deep well microplates (6 mL Storage Plate, Thermo Scientific ABgene) filled with 4 ml of sterile water per well and covered with adhesive PCR sealing foil (Thermo Scientific ABgene). After 1 week of incubation in a tropical plant growth chamber (30 °C, 80% humidity, 16 h day/8 h night) all liquid was recovered from wells, filtered (8 μm) and freeze-dried. The lyophilized RE powder obtained from 3 biological repetitions (4 microplates each) was dissolved in deionized water. A 1× final concentration of RE was used for bacterial treatments.

The composition of RE in terms of amino acids, organic acids (including cafeic, ferulic and sinapic acids) and soluble sugars was determined as previously described [130, 131] using a GC-MS Thermo TSQ Quantum with the silica column (Phenomenex ZB-5MSi (30 m, I.D. 0.25 mm, 0.25 μm)) with the following conditions: 60 to 350 °C gradient temperature during a 31 min run with an injection temperature of 230 °C. The concentration of polyphenols was estimated by the Folin colorimetric method [132]. The composition of RE is given in Table 4.

### Bacterial strains and media

Bacterial strains (*B. phymatum* STM815 (BP), *C. taiwanensis* LMG19424 (CT), and *R. mesoamericanum* STM3625 (RM)) were routinely cultivated in YM culture medium [133] at 28 °C and stored at − 80 °C in 20% glycerol. For RNA-seq analysis, bacteria were grown in a modified minimal medium, VSG (mVSG) [134], containing potassium phosphate monobasic (2 g.L^− 1^), potassium phosphate dibasic (2 g.L^− 1^), calcium chloride (0.45 mM), iron(III), chloride (37 μM), sodium glutamate (1 g.L^− 1^), sodium succinate (2 g.L^− 1^) and vitamins (biotin 0.5 mg.L^− 1^, nicotinic acid 0.05 mg.L^− 1^, calcium pantothenate 0.05 mg.L^− 1^, pyridoxine 0.05 mg.L^− 1^, thiamine 0.05 mg.L^− 1^, myo-inositol 5 mg.L^− 1^). pH was adjusted to 6.8 and the medium was sterilized by autoclaving. BIOLOG analysis of substrate assimilation by the bacterial strains was performed according to the manufacturer (API GN20, Biolog Inc., Hayward, CA, USA).

### Sampling of bacteria for RNA-seq

For each bacterium six liquid pre-cultures were grown in 10 mL of mVSG medium till mid-exponential phase and used to inoculate (at OD_600nm_ of 0.1) six 1 L Erlenmeyer flasks containing 200 mL of mVSG medium. For the RE-induced condition, *M. pudica* root exudates were added after 1 h of growth (1X final concentration). Bacteria were cultivated for 25 h at 28 °C with shaking at 150 rpm, and were sampled at 1, 3, 4.5, 6, 9 and 12 h post inoculation (hpi). At every time point four replicates of each culture were sampled into tubes containing a volume of ice-cold stop buffer (5% phenol in ethanol as described in [135]), and centrifuged for 10 min at 4000 rpm at 4 °C. Supernatants were removed and cells were frozen in liquid nitrogen and stored at − 80 °C.

### RNA preparation and sequencing

The mid-exponential growth phase was chosen for RNA sequencing, corresponding to 4.5 hpi for BP and CT and to 6 hpi for RM. Total RNA was purified using the RiboPure™ kit (Ambion, Austin, USA) following the manufacturer’s recommendations, then treated twice with DNAse I (Ambion), precipitated with ethanol and resuspended in RNAse-free water. Nine μg of total RNA was subjected to two successive rRNA removal steps using the Microbe Express™ kit (Ambion) to finally obtain 1 μg of enriched messenger RNA. The quantity and quality of total and enriched RNA were estimated using a NanoDrop ND-1000 and Bioanalyser Agilent 2100 (RNA nanochip), respectively (Additional file 1: Figure S13). All total and enriched messenger RNA samples were characterized by RIN values ranging from 9 to 10 indicating high quality.

Eighteen RNA samples (3 bacteria × 2 conditions × 3 biological repetitions) were sent for synthesis of corresponding cDNA libraries and sequencing to GATC Biotech (Konstanz, Germany). cDNA libraries were prepared according to the manufacturer’s instructions (Illumina TruSeq™ RNA Preparation) and labelled to sequence 2 to 3 libraries per lane (at least 50 million reads per library, read length of 36 bp) on a Genome Analyzer II (Illumina). Raw data sequences were submitted to ArrayExpress (www.ebi.ac.uk/arrayexpress) under accession: E-MTAB-4144.

### Statistical and functional analysis of transcriptomic data

RNAseq reads were mapped to CoDing Sequences (CDS) of each genome (extracted from the Microscope annotation platform [136] using CLC-Genomics workbench v5 (CLC Bio, Aarhus, Denmark) with standard mapping options. Only unique mapped reads (UMR) were considered and used to calculate read counts per CDS. Ribosomal RNAs were discarded from next analyses. Estimates of variance-mean dependence in read counts per CDS data (RCD) from the three replicates for each condition, and tests for differential expression based on a model using the negative binomial distribution, were performed using DESeq (R module) [137, 138]. First, a normalization of read numbers for replicates in each condition was performed (by estimates of size factors in DESeq and normalization), then differential expression values (FoldChange) and statistical values (pVal and adjusted pVal) assessing the false discovery rate (FDR) were calculated. Linear regression between means of gene read counts by condition (basemeans) was used to control the bias of treatment effect. Scatter plots, drawn to show the dispersion of read count data for each bacterium, and R^2^ correlation coefficents (R^2^ of 0.861, 0.972 and 0.974 for BP, CT and RM, respectively), validated the robustness of our RNA manipulation (Additional file 1: Figure S14A). Box plots of transformed log10 (Read Counts) were used to compare the distributions of our RNAseq data between conditions and between strains and to visualize the effect of normalization.

RE-regulated genes were identified following a two step process. First, a statistical significance cut-off at pVal ≤0.01 was applied (see Additional file 1: Figure S14B, volcano plots of Log2 (Fold change) versus –Log10 (*P*-Values)). This pVal cut-off can be considered as necessary and sufficient to focus on differentially expressed genes [138], but given the high number of regulated genes we applied a second cut-off for fold change <− 1.5 (down-regulated) or > 1.5 (up-regulated).

For the functional analysis of our data, the analysis of differentially expressed CDS was coupled with a verification of their annotation (considering the context of surroundings regions) and detection of putative operons. The clustering of regulated genes into functional groups was performed using the Bioprocess classification in MicroScope [136], which is based on the Comprehensive Microbial Resource (CMR, http://cmr.jcvi.org) [139].

### Comparative genomics

Comparative genomics were performed using the Microscope Microbial Genome Annotation & Analysis Platform (MAGE, [140]). Best Bidirectional Hits (BBH) were used to establish the correspondence of orthologous and specific genes between genomes; and provides lists of orthologs between each pair on all three strains. Venn diagrams were used to represent the number of orthologs shared between each genome. The RCircos module (adapted from Circos software [141]) was used to represent shared regulated orthologs.

### qPCR analyses

The primers used for quantitative PCR are listed in Additional file 1: Figure S5. The *uppS* and *hisB* genes were used as reference genes [43]. For qPCR, 0.5 μg of total RNA was reverse-transcribed with 200 U of Super-Script II (Invitrogen) using random decamer primers (2.5 μM, Ambion) at 25 °C for 10 min and then at 43 °C for 1 h. qPCR were performed on the Stratagene MXP3005P system using Power SYBR green master mix (Applied Biosystems). PCR started with a 10 min incubation at 95 °C, followed by 40 cycles consisting of 30 s at 95 °C and 30 s at 60 °C and 1 min at 72 °C. Primer specificity and the formation of primer dimers were checked by dissociation curves. PCR efficiency was calculated using the linear regression method on the log (fluorescence)-per-cycle-number data using Stratagene MXpro software. Prior to performing correlation analyses, the data were tested for normality using the Shapiro–Wilk test [142]. Because the data were not normally distributed, correlation analyses were done with Spearman’s Rho test [143].

## Additional file

Additional file 1: Figure S1. Venn diagram of gene orthologs across the 3 bacteria. **Figure S2.** Symbiotic plasmids syntenies and differentially regulated genes. **Figure S3.** Summary of RNAseq results per replicate and condition. **Figure S4.** Comparison of pVal and Fold Change values on the number of DEG. **Figure S5.** Genes targeted 5 for qPCR (A) & primers used (B). **Figure S6.** Comparison of gene expression between RNAseq counts and qPCR. **Table S1.** Excel tables of all DEG data shared or specific. **Figure S8.** Myo-inositol catabolism operons in BP, RM and CT. **Figure S9.** Biolog GN20 API galleries results for BP, CT and RM. **Figure S10.** Annotation of the putative rhizobitoxine biosynthesis operon in BP. **Figure S11.** Up-regulated (A) and down-regulated (B) T6SS in *B. phymatum* (BP). **Figure S12.** T4SS of *B. phymatum* STM815. **Figure S13.** Elution profiles of *C. taiwanensis* RNA samples. **Figure S14.** Scatter plots (A) & Volcano plot (B) of RNAseq data. (ZIP 15222 kb)

## Abbreviations

B.: *Burkholderia*
Biop: Bioprocess
BP: *Burkholderia phymatum* STM815
C.: *Cupriavidus*
CDI: Contact Dependent growth Inhibition system
CDS: Coding sequence
CMR: Comprehensive Microbial Resource
CT: *Cupriavidus taiwanensis* LMG19424
DEG: Differentially expressed genes
EPS: Exopolysaccharide
FC: Fold change
Kb: kilobase
LPS: Lipopolysaccharide
M.: *Mimosa*
Mb: Megabase
Mpf: Mating pair formation
NF: Nod factor
PHB: Polyhydroxybutyrate
pSym: Symbiotic plasmid
R.: *Rhizobium*
RE: Root Exudates
RM: *Rhizobium mesoamericanum* STM3625
sv.: Symbiovar
T3SS: Type 3 secretion system
T4SS: Type 4 secretion system
T6SS: Type 6 secretion system
TA: Toxin-Antitoxin system
VSG: Vincent Succinate Glutamate
