# Supplementary material for: Transcriptomic profiling of Burkholderia phymatum STM815, Cupriavidus taiwanensis LMG19424 and Rhizobium mesoamericanum STM3625 in response to Mimosa pudica root exudates illuminates the molecular basis of their nodulation competitiveness and symbiotic evolutionary history
Source: BMC Genomics. 2018 Jan 30;19:105. doi: 10.1186/s12864-018-4487-2 (PMC5789663; doi:10.1186/s12864-018-4487-2)
Supplement: Additional file 1: Figure S1. — Venn diagram of gene orthologs across the 3 bacteria. Figure S2. Symbiotic plasmids syntenies and differentially regulated genes. Figure S3. Summary of RNAseq results per replicate and condition. Figure S4. Comparison of pVal and Fold Change values on the number of DEG. Figure S5. Genes targeted 5 for qPCR (A) & primers used (B). Figure S6. Comparison of gene expression between RNAseq counts and qPCR. Table S1. Excel tables of all DEG data shared or specific. Figure S8. Myo-inositol catabolism operons in BP, RM and CT. Figure S9. Biolog GN20 API galleries results for BP, CT and RM. Figure S10. Annotation of the putative rhizobitoxine biosynthesis operon in BP. Figure S11. Up-regulated (A) and down-regulated (B) T6SS in B. phymatum (BP). Figure S12. T4SS of B. phymatum STM815. Figure S13. Elution profiles of C. taiwanensis RNA samples. Figure S14. Scatter plots (A) & Volcano plot (B) of RNAseq data. (ZIP 15222 kb) [file 12864_2018_4487_MOESM1_ESM.zip › Supplementary material-revised.docx]

Supplementary Material

**Supplementary File S1**: Venn diagram of gene orthologs across the 3 bacteria. P2

**Supplementary File S2:** Symbiotic plasmids syntenies and differentially regulated genes P3

**Supplementary File S3**: Summary of RNAseq results per replicate and condition P4

**Supplementary File S4**: Comparison of pVal and Fold Change values on the number of DEG P5

**Supplementary File S5**: Genes targeted 5 for qPCR (A) & primers used (B) P6-9

**Supplementary File S6.** Comparison of gene expression between RNAseq counts and qPCR P10

**Supplementary File S7**. Excel tables of all DEG data shared or specific Separate File

**Supplementary File S8**. Myo-inositol catabolism operons in BP, RM and CT P11

**Supplementary File S9**. Biolog GN20 API galleries results for BP, CT and RM P12-13

**Supplementary File S10**. Annotation of the putative rhizobitoxine biosynthesis operon in BP P14

**Supplementary File S11.** Upregulated (A) and downregulated (B) T6SS in *B. phymatum* (BP) P15-18

**Supplementary File S12**. T4SS of *B. phymatum* STM815 P19-20

**Supplementary File S13**: Elution profiles of *C. taiwanensis* RNA samples P21

**Supplementary File S14**: Scatter plots (A) & Volcano plot (B) of RNAseq data P22-23

Supplementary File S1: Venn diagram of gene orthologs across the 3 bacteria. Orthologs were obtained using the phyloprofile tool from Microscope software.

Supplementary File S2: Symbiotic plasmids syntenies and differentially regulated genes

Plasmids were aligned using the phyloprofile synteny tool of the Microscope plateform, with parameters 40% identity on at least 80% of protein length. Red and green lines indicates gene orthology in the same strand (red) or on the reverse strand (green). The fold change (FC) of upregulated genes by root exudates is indicated by red histograms under each rhizobial genome. Presence of Insertion sequences (IS) is indicated by pink bars. The nodulation gene region in each genome is indicated by a blue square.

Supplementary File S3. Summary of RNAseq results.

| Rhizobium | Sample ^a^ | total RNA nr of reads (millions) | Unmapped reads  (%) | rRNA  nr of reads (millions) | mRNA  nr of reads (millions) | mRNA  (%) |
| --- | --- | --- | --- | --- | --- | --- |
| BP - STM815 | Control 1 | 111 | 0.28 | 103.6 | 7.1 | 6.4 |
|  | Control 2 | 70 | 0.40 | 63.0 | 6.8 | 9.6 |
|  | Control 3 | 97 | 0.35 | 89.4 | 7.6 | 7.8 |
|  | Induced 1 | 69 | 0.33 | 66.0 | 3.5 | 5.0 |
|  | Induced 2 | 74 | 0.55 | 55.0 | 18.4 | 24.7 |
|  | Induced 3 | 55 | 0.54 | 41.1 | 13.9 | 24.9 |
|  |  |  |  |  |  |  |
| CT - LMG19424 | Control 1 | 57 | 0.31 | 47.8 | 9.1 | 16.0 |
|  | Control 2 | 98 | 0.35 | 85.0 | 12.8 | 13.2 |
|  | Control 3 | 82 | 0.22 | 68.6 | 13.3 | 16.3 |
|  | Induced 1 | 54 | 0.56 | 46.0 | 7.8 | 14.5 |
|  | Induced 2 | 62 | 0.64 | 53.9 | 8.0 | 12.9 |
|  | Induced 3 | 74 | 0.46 | 63.5 | 10.2 | 13.9 |
|  |  |  |  |  |  |  |
| RM - STM3625 | Control 1 | 42 | 1.62 | 30.08 | 10.5 | 26.0 |
|  | Control 2 | 69 | 1.78 | 55.7 | 11.5 | 17.1 |
|  | Control 3 | 34 | 1.42 | 22.8 | 9.7 | 30.0 |
|  | Induced 1 | 42 | 1.70 | 29.8 | 11.3 | 27.6 |
|  | Induced 2 | 39 | 1.84 | 27.8 | 10.1 | 26.7 |
|  | Induced 3 | 44 | 1.62 | 32.3 | 10.6 | 24.7 |

^a^: Control – RNA isolated from bacteria cultivated in absence of root exudates, Induced - RNA isolated from bacteria induced with root exudates,

Supplementary File S4. Comparison of pVal and Fold Change values on the number of differentially expressed genes (DEG) in BP (*B. phymatum*, CT (*C. taiwanensis*) and RM (*R. mesoamericanum*). The arrows in blue indicate the percentage of kept genes after pVal curing. Orange arrows indicate the percentage of kept genes after the application of the Fold Change limit. Yellow cases highlight the cut-off values retained for our analysis.

Supplementary File S5. Genes targeted 5 for qPCR (A) & primers used (B)

A) Choice of specific and orthologous genes for analysis by qPCR and their FoldChange observed in RNAseq data.

| **Specific /ortholog genes** | **BP** | **CT** | **RM** |
| --- | --- | --- | --- |
| RND-efflux  Stress response (efflux systems, shock proteins) | BURPHP2_0568  **4.86x** | _ | _ |
| Endoglucanase | BURPHP2_0639  **19.0x** | _ | _ |
| Rhizobitoxin, aminotransferase,  *rtxA*-like | BURPHP2_0646  **2.8x** | _ | _ |
| ACC deaminase  ***acdS*** | BURPHP2_0697  **2.2x** | _ | _ |
| ***iaaM*** | BURPHP2_0632  1.8x | _ | _ |
| non flagellar T3SS conserved transmembrane protein  ***sctV*** | *_* | RALTA_B1262  0.52x | _ |
| RTX-toxin  T1SS, Outer membrane efflux protein | _ | pRALTA_0238  0.59x | _ |
| multidrug efflux system, subunit A (RND family)  ***mdtA*** | _ | RALTA_A0540  1.71x | _ |
| ***bacA***  undecaprenyl pyrophosphate phosphatase; bacitracin resistance | _ | RALTA_A1644  0.2x | _ |
| ***copD***  Copper resistance protein D | _ | RALTA_B1965  **7.9x** | _ |
| ***norA***  NO-responding regulator | _ | RALTA_B2088  **6.3x** | _ |
| isocitrate lyase; glyoxylate cycle  ***aceA1*** | _ | RALTA_A1766  **11.0x** | _ |
| CopB ATPase, Copper resistance protein B precursor  ***copB*** | _ | RALTA_B1967  **11x** | _ |
| CopA Copper-resistance transmembrane precursor,  ***copA*** | _ | RALTA_B1968  **8,9x** | _ |
| D-amino acid dehydrogenase subunit  ***dadA1*** | _ | RALTA_A0798  **5x** | _ |
| NodJ membrane transport nodulation protein, ABC-type ***nodJ*** | _ | pRALTA_0360  **4x** | _ |
| conserved exported protein of unknown function | _ | _ | BN77v1_p11108F  **5.5x** |
| putative short-chain dehydrogenase/reductase | _ | _ | RHI36v1_p11608  **6.8x** |
| methyl-galactoside transporter subunit ; periplasmic-binding component of ABC superfamily  ***mglB*** | _ | _ | BN77v1_0783  **4.8x** |
| fructose ABC transporter, permease protein  ***frcC*** | _ | _ | BN77v1_1337  **3.4x** |
| ***nodO1*** | _ | _ | BN77v2_p290009  1.4x |
| ***nodO2*** | _ | _ | BN77v2_p2180035  1.8x |
| putative RTX toxins and related Ca2+-binding proteins, RTXtox | _ | _ | BN77v2_p2120002  **2.4x** |
| ***nodA*** | BURPHP2_0573  **14.0x** | pRALTA_0357  **5.2x** | nodA1(BN77v2_p280014)  1.98x  nodA2(BN77v2_p280039)  1x |
| ***nodB*** | BURPHP2_0581  **21.0x** | pRALTA_0363  **5.6x** | BN77v2_p280013  1.8x |
| ***nodC*** | BURPHP2_0578  **14.0x** | (pRALTA_0362)  **5.1x** | BN77v2_p280012  1.5x |
| ***nodD*** | BURPHP2_0582  1.8x | pRALTA_0364  0.95x | *nodD1* (BN77v2_p280016)  1.2x  *nodD3* (BN77v2_p2140045)  1.3x |
| ***uppS*** | BURPHK1_1440  0.7x | RALTA_A1687  0.96x | BN77v1_2711  0.94x |
| imidasol-glycerol-phosphate dehydrogenase (IGPD)  ***hisB*** | BURPHK1_2974  0.9x | RALTA_A2873  0.9x | BN77v1_0873  0.91 |
| **Fatty acid hydroxylase** | BURPHP2_0638  **27.0x** | pRALTA_0471  **2.4x** | BN77v2_p2140043  1.74x |
| ***flgG*** | BURPHK1_3188  1.7x | RALTA_B0282  **2.4x** | BN77v1_1547  **2.1x** |
| heavy-metal translocating P ATPase  ***copA*** | BURPHK2_1338  1.0x | RALTA_A3123  1.8x | BN77v1_3914  1.3x |
| put. branched chain aa ABC transporter  **livG2** | BURPHK1_0634  0.4x | RALTA_A2501  0.8x | BN77v1_p11120  0.6x |
| 50S ribosomal subunit proy L3  ***rplC*** | BURPHK1_3058  0.4x | RALTA_A2944  0.6x | BN77v1_2310  1.06x |
| sn-glycerol-3-phosphate-dehydrogenase  ***glpD*** | BURPHK1_0448  **6.0x** | RALTA_A2011  **3.5x** | BN77v1_4265  1.03x |

BP: *B. phymatum*STM815, CT: *C. taiwanensis* LMG19424, RM: *R. mesoamericanum* STM3625. Gene identifiers are the same as in NCBI. Numbers below gene identifiers are the fold change

B) Primers used for relative expression analysis of genes using qPCR

| Rhizobium | Gene | Primer number | Primer name | Sequence |
| --- | --- | --- | --- | --- |
| BP |  |  |  |  |
|  | *ispU=uppS* | 2389 | BURPHK1_1440-ispUf | ACATCGCGATCATCATGGAC |
|  |  | 2390 | BURPHK1_1440-ispUr | GGCGAACAGCGTCAGATATT |
|  | *rpoD* | 2788 | BURK2_-rpoD-1384F | CTGATGAAGGCGGTGGACAAG |
|  |  | 2789 | BURK2_-rpoD-1384R | GCGAGGACGGAGGGATGC |
|  | *nodA* | 2395 | BURPHP2_0573-nodAf | AGCCGAATTGGGACTGTATG |
|  |  | 2396 | BURPHP2_0573-nodAr | CTTTAAGGCATGCCGGACT |
|  | *nodB* | 2397 | BURPHP2_0581-nodBf | AACAGACACGGCGTGAGATA |
|  |  | 2398 | BURPHP2_0581-nodBr | CAATCCTGCCTTTGTCGATT |
|  | *nodC* | 2399 | BURPHP2_0578-nodCf | AGTGACTTTGGCGAAGATCG |
|  |  | 2400 | BURPHP2_0578-nodCr | TGCCCAACGTAGTTGTTGAC |
|  | *nodD* | 2792 | BURP2-nodD-94F | TTGAGTCAACCTGCCATGAGTGC |
|  |  | 2793 | BURP2-nodD-305R | CAGACCCTAAAGCGGCGATTCG |
|  | *st. desat* | 3682 | BP-qPCR-stdesF1 | CCTACCACTCACGCTGATTCAC |
|  |  | 3683 | BP-qPCR-stdesR1 | GTCCCAGATAGTCGTAAAGAACCC |
|  | *endogluc* | 4101 | BPendogluc_0639F | AACCTCGTCCTGCTTCTCG |
|  |  | 4102 | BPendogluc_0639R | TGATGCCGCTGGTGTTGG |
|  | *P2_0568* | 4103 | BP_RND-eff_0568F | GCGTTGTCGGCAAACATCGTG |
|  |  | 4104 | BP_RND-eff_0568R | CGAGCGTTTCCTGCGAGAGC |
|  | *P2_0646* | 4105 | BPrhizobit_0646F | CGGGTGTGTCGTTTGTAATCG |
|  |  | 4106 | BPrhizobit_0646R | TGGTCAGTCGCTCAAGTGC |
|  | *acdS* | 4107 | BP_ACCdeam_0697F | TTCGGCGGCAACAAGACC |
|  |  | 4108 | BP_ACCdeam_0697R | CGTAGTAGTTGACCCAGTTCTCC |
|  | *iaaM* | 4109 | BPiaaM_0632_F | CGCTCCCTTCCTTCAACTTTC |
|  |  | 4110 | BPiaaM_0632_R | GACCAAATTCTTCCTCCAATGC |
|  | *flgG* | 4111 | BPflgG_K1_3188_F | GCAGCAGACGGGCAACTC |
|  |  | 4112 | BPflgG_K1_3188_R | CGGGCAGCACCTGATAGC |
|  | *copA* | 4113 | BPcopA_K2_1338_F | TCGTCCAGTTCGTGTTCGG |
|  |  | 4114 | BPcopA_K2_1338_R | GCACCAGCGTAATCACCAC |
|  | *livG* | 4115 | BP_livG_K1_0634F | CGGTCACCAGCGGCGTCTC |
|  |  | 4116 | BP_livG_K1_0634R | GCCCATCACCAGTTTCACATCG |
|  | *rplC* | 4117 | BP_rplC_K1_3058F | CGCATCGCACGGTAACTC |
|  |  | 4118 | BP_rplC_K1_3058R | ACCCTTGACCAGCAGCAG |
|  | *glpD* | 4419 | BP_glpD_K1_0448F | TCCTCACACGCACGAAGC |
|  |  | 4120 | BP_glpD_K1_0448R | GCCGCACGCTGTAATTCG |
|  | *hisB* | 4121 | BP_hisB_K1-2974F | CGCTACGGTCATTCCTACG |
|  |  | 4122 | BP_hisB_K1-2974R | CCGCATGATTGACGAAACC |
| CT |  |  |  |  |
|  | *uppS* | 2629 | STM894uppS_245F | CGTTCCTGATGCGGCTGTTC |
|  |  | 2714 | STM894uppS_432R | GTAGTTGGCGGCGATGGTG |
|  | *rpoD* | 2717 | STM894rpoD2_517F | GCCTGCCCGTTCACCATC |
|  |  | 2718 | STM894rpoD2_709R | CGCTGTCGTCGTCATCTTCG |
|  | *nodD* | 2676 | STM894nodD_20F | ACCTGAACCTTCTCGTCGCACTTG |
|  |  | 2677 | STM894nodD_181R | CGAACTCCCGCCCACGCATAAC |
|  | *nodA* | 2625 | STM894nodA_348F | GCTTCGCATACCGTTCGCTTTC |
|  |  | 2626 | STM894nodA_527R | GGCATAACCACGACGAGGACATC |
|  | *nodB* | 2619 | STM894nodB_440F | CGCTTCACTGGTCGGTAG |
|  |  | 2620 | STM894nodB_612R | CCTCACGGTTTGCTCTCG |
|  | *st. desat* | 3684 | CT-qPCR-stdesF1 | CCTACCACTCACGCTGATTCAC |
|  |  | 3685 | CT-qPCR-stdesR1 | GTCCCAGATAGTCGTAAAGAACCC |
|  | *B1262 T3SS sctV* | 4141 | CT_T3SS-sctV_F | CCGTCGCTGCTGGTCTCC |
|  |  | 4142 | CT_T3SS-sctV_R | GGAACACCGCAATGGCAAGG |
|  | *0238* | 4143 | CT_RTX-toxin_F | AGTCTCGCCGCTGCTCCTG |
|  |  | 4144 | CT_RTX-toxin_R | TGCCTGGTCACATCCGTCCTC |
|  | *mdtA* | 4145 | CT_mdtA_F | ACGAAGCCAGCAAGGTCAAGG |
|  |  | 4146 | CT_mdtA_R | GCACCGCTCGCACCATCG |
|  | *flgG* | 4147 | CT_flgG_F | GCACCACCGCCTATACCC |
|  |  | 4148 | CT_flgG_R | TCTCGCCCATGCTTTCCAG |
|  | *copA2* | 4149 | CT_copA2_F | TACAAGGCGGGCTGGAAG |
|  |  | 4150 | CT_copA2_R | ATGGCATCGGCAGTCTGG |
|  | *livG2* | 4151 | CT_livG2_F | GTCGGCATCGGCAAGTAC |
|  |  | 4152 | CT_livG2_R | CCATCGTTCTTGATCTTGTCG |
|  | *rplC* | 4153 | CT_rplC_F | GCAGGGCGTGACCATCGG |
|  |  | 4154 | CT_rplC_R | ACGGTGCGGGTGACATCG |
|  | *glpD* | 4155 | CT_glpD_F | CTCGGCGGCGGCACCTAC |
|  |  | 4156 | CT_glpD_R | TCCACGGCACCACGAAGAGC |
|  | *hisB* | 4157 | CT_hisB_F | GCGACAAGAAGGGCATCAC |
|  |  | 4158 | CT_hisB_R | CGGCATGGTTGACGAAGC |
| RM |  |  |  |  |
|  | *rpoD* | 2813 | RH3714rpoD756F | GGAAGAAGAAGACGAGGACGAATC |
|  |  | 2814 | RH3714rpoD983R | GACTTGACCGCCTTGATGAGC |
|  | *uppS* | 2815 | RH2711uppS309F | CGACCGCCATAGTCTCAAGAGC |
|  |  | 2816 | RH2711uppS535R | GGATGCCTGCCGTGTCAAGC |
|  | *nodA1* | 2796 | RHp20307nodA94F | GGACCCACAGGAACATTCAACG |
|  |  | 2797 | RHp20307nodA311R | TGGCTGATACCGAGACCCTTC |
|  | *nodA2* | 2794 | RHp20307nodA292F | GAGGGTTTGGGGATAGGACATTCG |
|  |  | 2795 | RHp20307nodA493R | GCGTGGGCGGCAAGTCAG |
|  | *nodB* | 2798 | RHp20195nodB_296F | TCACGACTGGTAGCACAATCTCTG |
|  |  | 2799 | RHp20195-nodB_487R | CGAGTACCGCAGTGACGATGG |
|  | *nodC* | 2800 | RHp20194nodC423F | AGACACCATCCTTGAGCCTGAG |
|  |  | 2801 | RHp20194nodC633R | GCACGGACCGCAGCAACAC |
|  | *nodD1* | 2809 | RHp20198nodD182F | CGCAGCATCCACCTAAGCCAACC |
|  |  | 2810 | RH20198nodD1299R | CGGAAGCGGCGATCTGACTTAACC |
|  | *nodD3* | 2811 | RHp20313nodD380F | CGCACAGCATCAATCTCAGTCAG |
|  |  | 2812 | RHp20313nodD3360R | CGCCACACGCTCCACAAC |
|  | *st. desat* | 3686 | RM-qPCR-stdesF1 | GCACTTCTACGCTTTGGGTTTG |
|  |  | 3687 | RM-qPCR-stdesR1 | AATTCCACGCCGTCAGTTCG |
|  | p290009 *nodO1* | 4123 | RM_nodO1_F | CGACGAGGGCAACGACTAC |
|  |  | 4124 | RM_nodO1_R | GGAACTGGAACGCAAAGGTG |
|  | p2180035 *nodO2* | 4125 | RM_nodO2_F | GGTAGGGATTCGTTGGCAGGAG |
|  |  | 4126 | RM_nodO2_R | TGAAGGTCGGCGTGATTGATAAAG |
|  | p2120002 | 4127 | RM_RTXtox_F | CGGAGCAGACACTTTTCACTTC |
|  |  | 4128 | RM_RTXtox_R | TCCCTCGGTCGCAATTACG |
|  | *flgG* | 4129 | RM_flgG_F | GCACATCCAGGGCGAACTTG |
|  |  | 4130 | RM_flgG_R | GAGCCAGTCGGAATCGTGATG |
|  | *copA* | 4131 | RM_copA_F | AGAACGAGCCACGCCATCAAG |
|  |  | 4132 | RM_copA_R | TCACGACCAGACCATCAACAGG |
|  | *livG* | 4133 | RM_livG_F | CCGAACCTTCCAGAATCTTGCC |
|  |  | 4134 | RM_livG_R | ATGATTTGCCGTGCTCGTTCC |
|  | *rplC* | 4135 | RM_rplC_F | GGCACGGCGAAGGTCAAG |
|  |  | 4136 | RM_rplC_R | GCCCGCAAAGCCCTTACC |
|  | *glpD* | 4137 | RM_glpD_F | CGCTTCGTGCTGCCTTATC |
|  |  | 4138 | RM_glpD_R | ACCCAACCGTCCGAGTATTC |
|  | *hisB* | 4139 | RM_hisB_F | CGAGACTTCCGTTTCCGTTTCC |
|  |  | 4140 | RM_hisB_F | CGATGCGTAGCGTGTGATGC |
|  | p11608 | 4453 | RN77v1_p11608_F | TGGGAAAGCGAGTTGGAAC |
|  |  | 4454 | RN77v1_p11608_R | TGAGCGTCATAGGTGATTGC |
|  | *mglB* | 4455 | mglB_F | GCAATCATCGTCAACCCAGTC |
|  |  | 4456 | mglB_R | GGAGAGTTCGCCCATCAGG |
|  | *frcC* | 4457 | frcC_F | ACTTCATCTATTCCGCCAAC |
|  |  | 4458 | frcC_R | CCGTGTGATTGAGGATATACC |

BP: *B. phymatum*STM815, CT: *C. taiwanensis* LMG19424, RM: *R. mesoamericanum* STM3625,

f : forward, r : reverse,

*^a^, ispU* : Undecaprenyl pyrophosphate synthetase (UPP synthetase) ; *pdhC :* Dihydrolipoamide acétyltransférase; *rpoD:* facteur Sigma-70; *nodA:* acétyltransférase NodA; *nodB:* polysaccharide desacetylase; *nodC:* glycosyl transferase; *nodD:* proteine D de nodulation;

Supplementary File S6. Comparison of gene expression between RNAseq counts (y axis) and qPCR expression (x axis) normalised either with uppS (blue dots) or hisB green triangles.

Supplementary File S8 (A,B,C). Myo-inositol catabolism operons in BP, RM and CT

A: Putative ortholog operons in BP, RM and RL (R. leguminosarum bv viciae), induced in BP and RM. It was already described in RL as a myo-inositol catabolism (transfer and metabolism) system distributed between 3 loci (Summers et al., 1999).; B: percentage of nucleotide identity between ortholog genes; C: gene description, their FC and level of expression (in number of reads per CDS).

**Supplementary File S9.** API GN20 Galeries tests for BP, CT and RM.

The capacity of each strain to use several carbon sources was tested on GN20 Biolog tests, at 24 and 48H post inoculation in Biolog microplates. Legend: blank = no growth, +/-: very low growth; +: low growth; ++: medium growth; +++:high growth.

|  | BP_24h | BP_48h | CT_24h | CT_48h | RM_24h | RM_48h |
| --- | --- | --- | --- | --- | --- | --- |
| Water |  |  |  |  |  |  |
| α-Cyclodextrin |  |  |  |  |  |  |
| Dextrin | +/- |  |  |  | + |  |
| Glycogen |  |  |  |  |  |  |
| Tween 40 | +++ | +++ |  | +++ |  |  |
| Tween 80 | +++ | +++ |  | +++ |  |  |
| N-Acetyl-Dgalactosamine |  |  |  |  | + | ++ |
| N-Acetyl-Dglucosamine | +++ | +++ |  |  | + | ++ |
| Adonitol | ++ | +++ |  |  | + | ++ |
| L-Arabinose | ++ | +++ |  |  | + | ++ |
| D-Arabitol | ++ | +++ |  |  | + | ++ |
| D-Cellobiose |  |  |  |  | + | ++ |
| i-Erythritol |  |  |  |  |  |  |
| D-Fructose | ++ | ++ |  |  | + | ++ |
| L-Fucose | + | ++ |  |  | + | ++ |
| D-Galactose | +++ | ++ |  |  | + | ++ |
| Gentiobiose |  |  |  |  | ++ | ++ |
| α-D-Glucose | +++ | +++ |  |  | ++ | ++ |
| m-Inositol | ++ | +++ |  |  | ++ | ++ |
| α-D-Lactose |  |  |  |  | ++ | ++ |
| Lactulose |  |  |  |  | ++ | ++ |
| Maltose |  |  |  |  | ++ | ++ |
| D-Mannitol | +++ | +++ |  |  | ++ | ++ |
| D-Mannose | +++ | +++ |  |  | ++ | ++ |
| D-Melibiose |  |  |  |  | + | ++ |
| β-Methyl-D-Glucoside |  |  |  |  | + | ++ |
| D-Psicose | +/- |  |  |  | +/- | +/- |
| D-Raffinose | + | + |  |  | +/- | + |
| L-Rhamnose | ++ | +++ |  |  | + | ++ |
| D-Sorbitol | ++ | +++ |  |  | ++ | ++ |
| Sucrose | +++ | +++ |  |  | ++ | ++ |
| D-Trehalose |  | +/- |  |  | ++ | ++ |
| Turanose |  |  |  |  | ++ | ++ |
| Xylitol |  |  |  |  |  | + |
| Methyl Pyruvate | +++ | +++ | ++ | ++ | + | ++ |
| Mono-Methyl-Succinate | + |  |  | +++ |  | +/- |
| Acetic Acid | ++ | + | +++ | +++ | + | ++ |
| Cis-Aconitic Acid | +++ | +++ | +++ | +++ | ++ | ++ |
| Citric Acid | +++ | +++ | +++ | +++ | + | + |
| Formic Acid | ++ | ++ | +++ | +++ | +++ | +++ |
| D-Galactonic Acid Lactone |  | +/- |  |  |  |  |
| D-Galacturonic Acid | +++ | +++ |  |  |  |  |
| D-Gluconic Acid | +++ | +++ |  | +/- |  |  |
| D-Glucosaminic Acid | ++ | +++ |  |  | + | ++ |
| D-Glucuronic Acid | +++ | +++ |  |  |  |  |
| α-Hydroxy Butyric Acid |  |  | + | +/- |  |  |
| β-Hydroxy Butyric Acid | +++ | +++ | +++ | +++ | + | ++ |
| γ-Hydroxy Butyric Acid |  |  |  |  |  |  |
| p-Hydroxy Phenylacetic Acid | + | +++ | ++ | +++ |  |  |
| Itaconic Acid |  |  | +/- | + |  |  |
| α-Keto Butyric Acid | + |  | +/- | +/- |  |  |
| α-Keto Glutaric Acid |  |  |  |  |  |  |
| α-Keto Valeric Acid |  | +/- |  | + |  |  |
| D,L-Lactic Acid | +++ | +++ | +++ | +++ | +/- | + |
| Malonic Acid |  | +/- |  |  |  |  |
| Propionic Acid |  | +/- | +++ | +++ |  |  |
| Quinic Acid | +++ | +++ |  |  | + | + |
| D-Saccharic Acid | +++ | +++ |  |  |  |  |
| Sebacic Acid |  |  | +++ | +++ |  |  |
| Succinic Acid | +++ | +++ | +++ | +++ | + | + |
| Bromo Succinic Acid | +++ | +++ | +++ | +++ | + | ++ |
| Succinamic Acid |  |  | +++ | +++ |  |  |
| Glucuronamide |  |  |  |  |  |  |
| L-Alaninamide |  |  |  | +/- |  |  |
| D-Alanine | +/- | +/- | ++ | +++ |  |  |
| L-Alanine |  | +/- | ++ | +++ | +/- | + |
| L-Alanylglycine |  | +/- |  |  |  |  |
| L-Asparagine | +++ | +++ | +++ | +++ |  |  |
| L-Aspartic Acid | +++ | +++ | +++ | +++ |  |  |
| L-Glutamic Acid | +++ | +++ | +++ | +++ | + | + |
| Glycyl-Laspartic Acid |  |  |  |  |  |  |
| Glycyl-Lglutamic Acid |  |  |  |  |  |  |
| L-Histidine | ++ | +++ | +++ | +++ | ++ | +++ |
| Hydroxy-LProline | + | + |  |  | + | ++ |
| L-Leucine | ++ | ++ | ++ | +++ |  |  |
| L-Ornithine |  | +/- |  |  |  | +/- |
| LPhenylalanine | + | ++ | ++ | +++ |  |  |
| L-Proline | ++ | +++ | ++ | +++ | +/- | ++ |
| L-Pyroglutamic Acid | ++ | +++ | ++ | +++ |  |  |
| D-Serine |  | +/- |  |  |  |  |
| L-Serine | +/- | + |  | ++ |  | +/- |
| L-Threonine |  | +/- |  | ++ |  |  |
| D,L-Carnitine |  | + |  |  | + | +++ |
| γ-Amino Butyric Acid | ++ | +++ |  | +/- |  | +/- |
| Urocanic Acid |  |  | +++ | +++ | +/- | ++ |
| Inosine |  |  |  |  |  |  |
| Uridine |  |  |  |  |  | +/- |
| Thymidine |  |  |  |  |  |  |
| Phenyethylamine |  |  |  |  |  |  |
| Putrescine |  |  |  |  |  |  |
| 2-Aminoethanol | ++ | +++ |  |  |  |  |
| 2,3-Butanediol |  |  |  |  |  |  |
| Glycerol | +/- | ++ |  |  | + | +++ |
| D,L-α-Glycerol Phosphate |  | +/- |  |  |  |  |
| Glucose-1-Phosphate |  |  |  |  |  |  |
| Glucose-6-Phosphate | ++ | +++ |  |  |  |  |

**Supplementary File S10**. Annotation of the putative rhizobitoxine biosynthesis operon in BP, and comparison with operon described in *Bradyrhizobium elkanii (*Yasuta et al., 2001; Sugawara et al., 2007).

**Supplementary Figure S11**. Comparison of T6SS of *B. phymatum* STM815 (BURPHP1_623-0645) with the closest T6SS of 5 other *Burkholderia* species. A: comparison of operons architecture, B: Table showing FC of T6SS induced with RE and percentage identity (% Id) of corresponding genes from 3 closest operons depicted in A, C: syntenic clusters T6SS present in other bacterial genomes compared to BP T6SS (numbers in red describe % if genes identity in comparison to the corresponding BP genes). **BP**: *B. phymatum* STM815; **BPf** : *B. phytofirmans* PsJN; **BMa**: *B. mallei* ATCC 23344; **Bpma**: *B. pseudomallei* K96243; **BG**: *B. gladioli* BSR3; **Bsp** : *Burkholderia* sp. TJI49.

**Supplementary Figure 11B**. Comparison of T6SS of *B. phymatum* STM815 (BURPHP1_0479-0506) with the closest T6SS of 3 other *Burkholderia* species. A: comparison of operons architecture, B: Table showing FC of T6SS repressed in presence of RE and percentage identity (% Id) of corresponding genes from 3 closest operons depicted in A, C: syntenic clusters T6SS present in other bacterial genomes compared to BP T6SS (numbers in red describe % if genes identity in comparison to the corresponding BP genes). **BP**: *B. phymatum* STM815; **BTr**: *B. terrae* BS001; **BJP**: *Burkholderia* sp. JPY347; **BK**: *B. kururiensis* M130; **BTccg**: *Burkholderia* sp. CCGE1002.

**Supplementary File S12**. Comparison of T4SS of *B. phymatum* STM815 with the closest T4SS of 3 other *Burkholderia* species. A: comparison of operons architecture, B: Table showing FC of T4SS induced with RE and percentage identity (% Id) of corresponding genes from 3 closest operons depicted in A, C: syntenic clusters T4SS present in other bacterial genomes compared to BP T4SS (numbers in red describe % if genes identity in comparison to the corresponding BP genes). **BP**: *B. phymatum* STM815; **BPx**: *B. phenoliruptrix* BR3459a; **BTr**: *B. terrae* BS001; **BM**: *B. mimosarum* STM3621, Mpf: mating pore formation module; Dtr: DNA transfer and replication module.

**Supplementary File S13:** Elution profiles of *C. taiwanensis* RNA samples analysed on Bioanalyser (Agilent). Comparison of total RNA (in red), RNA enriched in mRNA after 1^st^ round of rRNA subtraction (in blue) and after 2^nd^ round of subtraction (in green) for the presence of rRNA 16S and 23S.

Supplementary File S14A. Dispersion of read counts data by Scatter plots

BaseMeanA/B = number of read counts per CDS from control (A) or RE-induced (B) condition.

Supplementary File S14B: Volcano plots of Log2 (Fold change) versus –Log10 (P-Values) (B
